# A high-quality genome assembly of *Morinda officinalis*, a famous native southern herb in the Lingnan region of southern China

**DOI:** 10.1038/s41438-021-00551-w

**Published:** 2021-06-01

**Authors:** Jihua Wang, Shiqiang Xu, Yu Mei, Shike Cai, Yan Gu, Minyang Sun, Zhan Liang, Yong Xiao, Muqing Zhang, Shaohai Yang

**Affiliations:** 1 Guangdong Provincial Key Laboratory of Crops Genetics & Improvement, Crops Research Institute, Guangdong Academy of Agricultural Sciences, 510640 Guangzhou, China; 2 DongFuhang High-tech Agricultural Planting and Management Co., Ltd, 526000 Zhaoqing, China; 3 Coconut Research Institute, Chinese Academy of Tropical Agricultural Sciences, 571339 Wenchang, China; 4 State Key Lab for Conservation and Utilization of Subtropical Agric-Biological Resources, Guangxi University, 530005 Nanning, China

**Keywords:** Genome evolution, Medical genomics, Genome duplication

## Abstract

*Morinda officinalis* is a well-known medicinal and edible plant that is widely cultivated in the Lingnan region of southern China. Its dried roots (called *bajitian* in traditional Chinese medicine) are broadly used to treat various diseases, such as impotence and rheumatism. Here, we report a high-quality chromosome-scale genome assembly of *M. officinalis* using Nanopore single-molecule sequencing and Hi-C technology. The assembled genome size was 484.85 Mb with a scaffold N50 of 40.97 Mb, and 90.77% of the assembled sequences were anchored on eleven pseudochromosomes. The genome includes 27,698 protein-coding genes, and most of the assemblies are repetitive sequences. Genome evolution analysis revealed that *M. officinalis* underwent core eudicot γ genome triplication events but no recent whole-genome duplication (WGD). Likewise, comparative genomic analysis showed no large-scale structural variation after species divergence between *M. officinalis* and *Coffea canephora*. Moreover, gene family analysis indicated that gene families associated with plant–pathogen interactions and sugar metabolism were significantly expanded in *M. officinalis*. Furthermore, we identified many candidate genes involved in the biosynthesis of major active components such as anthraquinones, iridoids and polysaccharides. In addition, we also found that the DHQS, GGPPS, TPS-Clin, TPS04, sacA, and UGDH gene families—which include the critical genes for active component biosynthesis—were expanded in *M. officinalis*. This study provides a valuable resource for understanding *M. officinalis* genome evolution and active component biosynthesis. This work will facilitate genetic improvement and molecular breeding of this commercially important plant.

## Introduction

**Fig. 1:**
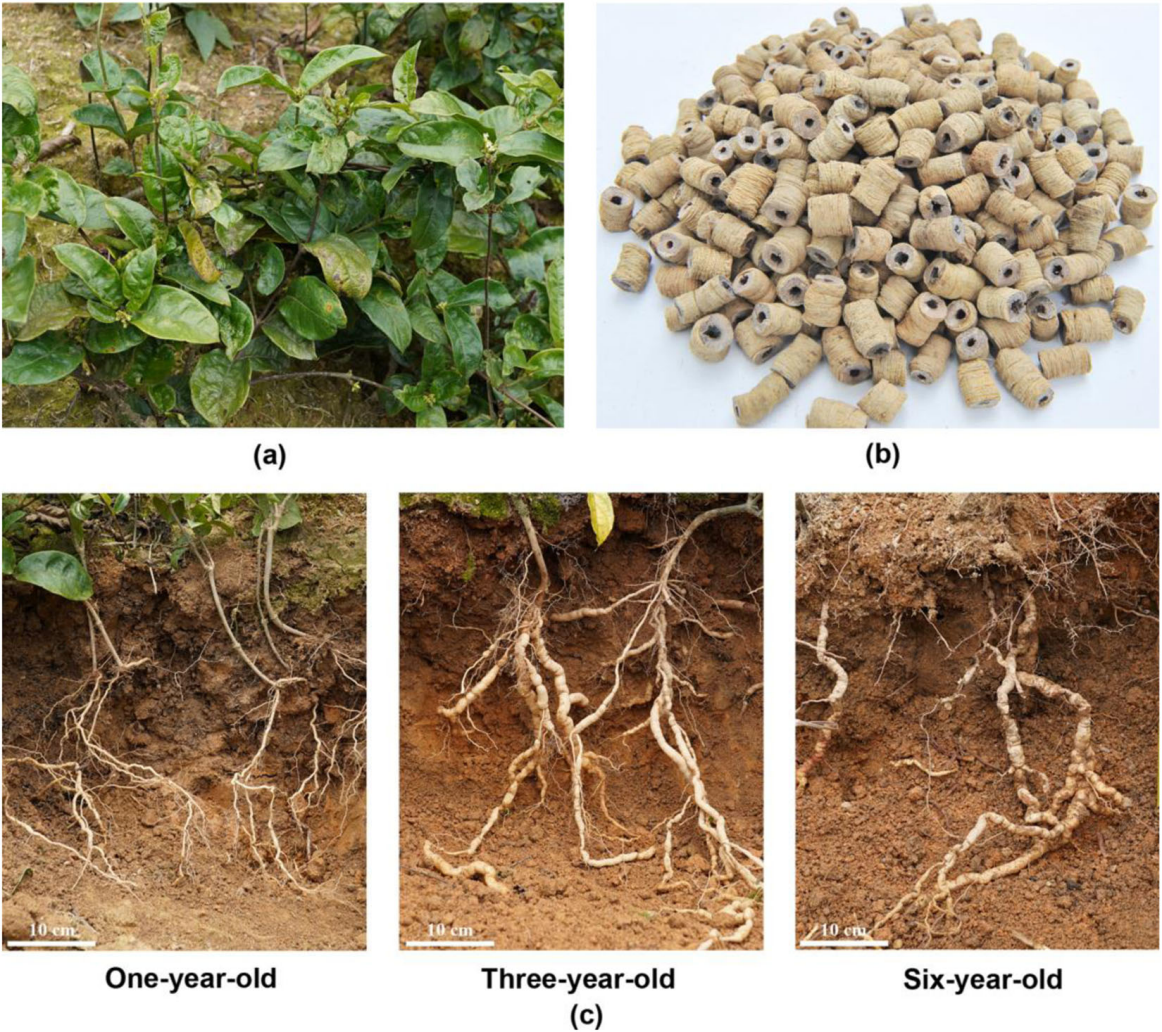
Morphological characteristics of *M. officinalis*. **a** The *M. officinalis* plant. **b** The roots of *M. officinalis* with the woody core removed (named *bajitian* in traditional Chinese medicine). **c** The roots of *M. officinalis* at different growth stages

*Morinda officinalis* How, belonging to the genus *Morinda* of the family Rubiaceae, is a perennial vine naturally distributed in southern China and northern Vietnam (Fig. 1a)^1^. *M. officinalis*, a commonly used traditional Chinese medicinal plant, was first reported in Shen Nong Ben Cao Jing and accepted in the Chinese Pharmacopoeia in 1963. The roots of *M. officinalis*, named *bajitian* in traditional Chinese medicine, are one of the four famous southern herbs from the Lingnan region of southern China. The roots of *M. officinalis* are widely used for the treatment of various diseases, such as impotence, infertility, abnormal menstruation, rheumatism, and arthralgia (Fig. 1b, c)^2,3^. In China and northeast Asia, *M. officinalis* is also usually used as a tonic for nourishing the kidneys and enhancing immune functioning in the body^2,4^.

Phytochemical studies have shown that *M. officinalis* contains anthraquinones, iridoids, flavonoids, polysaccharides, volatile oils, and other important compounds^5,6^. Anthraquinones are one of the main active components and mainly contain physcion, rubiadin-1-methylether, anthragallol-2-methylether, etc., which have various biological activities such as antibacterial, anticancer, anticoagulant, and antiviral activities^7,8^. Monotropein, a kind of iridoid compound, has potent antiinflammatory and analgesic effects and is the main component of *M. officinalis* for dispelling wind and eliminating dampness^9^. The polysaccharides in *M. officinalis* mainly consist of glucose and fructose, which have antifatigue, antidepressant, and antiosteoporosis roles^10,11^. Various pharmacological and clinical studies have linked *M. officinalis* with aphrodisiac and immunomodulatory effects and antiosteoporosis, antidepression and antiinflammatory properties^12–14^.

In China, Guangdong Province is the main planting area for *M. officinalis*, accounting for approximately 90% of its output. In this province, Gaoyao District and Deqing County (Zhaoqing City) are authentic *M. officinalis*-producing areas^15,16^. In recent years, wild resources of *M. officinalis* have been significantly threatened in China to a level risking extinction due to a sharp increase in *M. officinalis* market demand, especially in Guangdong Province^15^. Therefore, artificially cultivated *M. officinalis* has become the primary source of related medicinal materials in China. *M. officinalis* has a long cultivation period of approximately five years; moreover, its sexual reproduction cycle is complex. Long-term asexual reproduction of *M. officinalis* has led to germplasm depletion that seriously affects its quality and yield. Thus, diseases of *M. officinalis* are becoming a severe issue, especially stem rot caused by *Fusarium oxysporum*, which is devastating^17^. Thus, the breeding of promising new varieties is urgently needed.

The genomic information of *M. officinalis* can lay the foundation for improving the quality of these medicinal materials, accelerating molecular breeding, protecting wild resources, and aiding the discovery and utilization of functional genes^18^. Whole-genome sequencing has been performed for many medicinal plants, such as *Scutellaria baicalensis*, *Isatis indigotica*, weeping forsythia, and *Macleaya cordata*^19–22^. However, the genome sequence of *M. officinalis* has not yet been reported, which restricts the development of functional genomics and molecular breeding of this plant. In this study, we generated a high-quality genome for *M. officinalis* by nanopore sequencing and Hi-C technology to elucidate its genomic characteristics. Transcriptome sequencing was also carried out to identify the candidate genes related to active compound biosynthesis. The reference genome information obtained in this research will be a valuable resource for promoting genetic improvement and understanding the biosynthesis of active ingredients of this medicinal plant.

## Results

### Genome assembly and quality validation

To evaluate the genome size and heterozygosity of *M. officinalis*, a total of 61.4 Gb short reads from the MGISEQ-2000 sequencing platform were subjected to K-mer analysis (Supplementary Table S1). The 17-mer frequency curve showed a bimodal distribution, with the highest peak occurring at a depth of 54 (Supplementary Fig. S1a). Based on the total number of K-mers, the genome size and heterozygosity of *M. officinalis* were estimated to be 485.4 Mb and 1.32%, respectively (Supplementary Fig. S1b). These results indicated that the genome of *M. officinalis* was small but highly heterozygous.

**Fig. 2:**
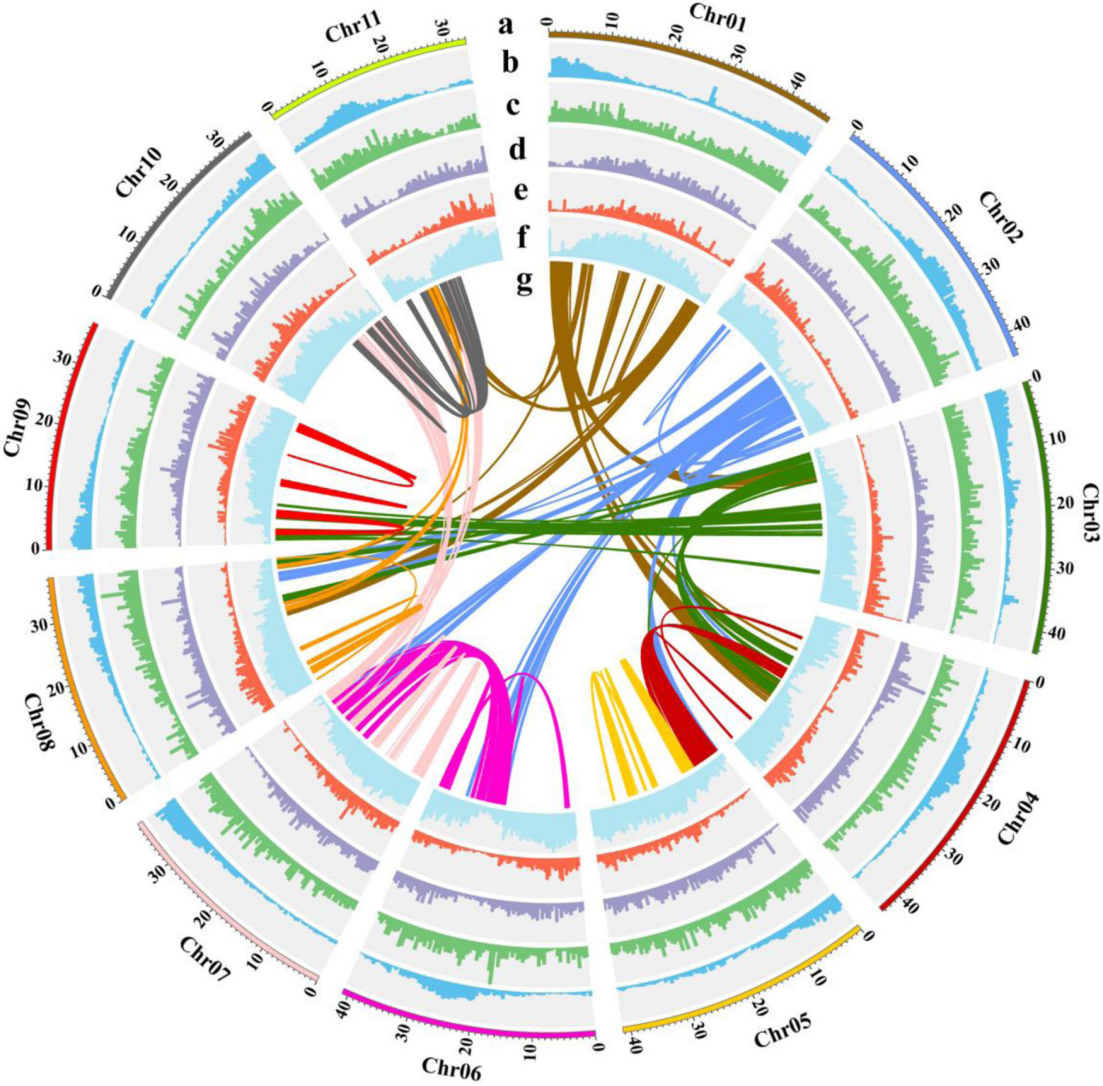
Characterization of the *M. officinalis* genome. The circle from outside to inside represents chromosomes (**a**), gene density (**b**), tandem repeat density (**c**), LTR-Copia density (**d**), LTR-Gypsy density (**e**), transposon element density (**f**), and gene collinearity connected by curved lines (**g**). All distributions are drawn in a window size of 1 Mb

*De novo* assembly of the 62.92 Gb of single-molecule long reads from the Oxford Nanopore PromethION sequencing platform was performed with NextDenovo software (Supplementary Table S1). After removing redundant and contaminated sequences (nontarget classes, mitochondria, and chloroplasts), the final postcorrection genome size was 484.85 Mb, with a contig N50 of 4.21 Mb (Table 1). The genome size was similar to that estimated by the genome survey. Employing Hi-C technology, 398.8 million clean reads from the Illumina NovaSeq 6000 sequencing platform were used for chromosome construction to further refine the *M. officinalis* genome assembly. Moreover, using the agglomerative hierarchical clustering method in LACHESIS software, a total of 99.94% of the assembly was anchored to 11 pseudochromosomes; the size ranged from 33.06 Mb to 47.00 Mb with a contig N50 of 3.61 Mb and scaffold N50 of 40.97 Mb (Table 1 and Supplementary Table S2). Finally, the contig sequences were connected in the determined order and direction by adding 100 N to obtain the final chromosome-level genome sequence with a chromosome mount rate of 90.77% (Fig. 2 and Supplementary Table S2). A Hi-C interaction heatmap showed that the clustering, ordering, and orientation of the contigs was valid, providing the first high-quality chromosome-scale genome assembly for *M. officinalis* (Supplementary Fig. S2).

**Table 1:** Summary of *M. officinalis* genome assembly and annotation

Items	Number	Size (bp)
Genome assembly		
Total contigs	209	484,851,740
Contig N50	32	4,213,846
Contig N90	118	1,061,677
Total scaffolds	64	484,869,040
Scaffold N50	6	40,972,926
Scaffold N90	11	33,060,658
Pseudochromosomes	11	440,084,418
Genome annotation		
Repetitive sequences	58.04%	281,412,890
Noncoding RNAs	2298	342,651
Protein-coding genes	27,102	101,967,209

A variety of methods have been employed to evaluate the assembly quality of genomes. Here, the assembly completeness was evaluated using Benchmarking Universal Single-Copy Orthologs (BUSCOs). The BUSCO analysis identified 97.02% of the complete BUSCOs in the assembly (Supplementary Table S3). To assess assembly consistency, the short reads were mapped to the genome using bwa software. In total, ~99.26% of the clean data were mapped to the genome assembly (Supplementary Table S4). The mapping ratio of the RNA-seq reads from different tissues was in the range of ~93.94–96.08% (Supplementary Table S5). We further evaluated potential contamination in the genome by using GC depth analysis. The GC depth scatter plot showed that the GC content was distributed at 30–40%, and the sequencing depth was concentrated at 110–150×, indicating high quality without contamination in the data (Supplementary Fig. S3). All these results indicated high completeness and consistency of the *M. officinalis* genome assembly.

### Genome annotation

Homology-based annotation and a *de novo* approach were applied to identify transposable elements (TEs) and tandem repeats in the *M. officinalis* genome. In total, we identified 281.41 Mb of nonredundant repetitive sequences, accounting for 58.04% of the assembled genome (Supplementary Table S6). Of these predicted repeats, TEs comprised the largest proportion (54.18%), including 42.92% Class I repeats and 11.26% Class II repeats. Long terminal repeat (LTR) retrotransposons accounted for 35.79% of the genome (Supplementary Table S6). Based on the assembled genome, a total of 209 rRNAs, 207 snRNAs, 78 miRNAs, and 644 tRNAs were predicted (Supplementary Table S7).

A total of 27,102 nonredundant protein-coding genes were predicted by using a combination of *de novo*, homolog-based, and transcriptome-based predictions (Table 2). The average gene length and coding sequence size were 3762.35 and 1169.11 bp, respectively, with an average of five exons per gene (Table 2). Then, we performed functional annotation of predicted genes by comparison with different databases. Overall, 24,769 (91.39%) genes were functionally annotated in at least one of the public databases, and 6153 (22.70%) genes could be annotated in all databases (Supplementary Fig. S4a). A total of 24,637 (90.90%) genes showed homologous genes in the NR database, while 20,712 (76.42%) genes were similar to proteins in the SwissProt database. Among the blastx top hits, species of the genus *Coffea* (Rubiaceae family) showed the highest proportion (82.77%) of homologous genes, including *Coffea arabica* (49.54%), *Coffea eugenioides* (20.44%), and *C. canephora* (12.79%) (Supplementary Fig. S4b). In addition, 14,998 (55.34%) genes were assigned to at least one GO term and classified into 41 GO functional subcategories (Supplementary Fig. S4c). To further understand the metabolic pathways of *M. officinalis*, 9737 (35.93%) genes were annotated in the KEGG pathway database (Supplementary Fig. S4d). Pathways associated with “biosynthesis of other secondary metabolites” (466 genes) and “metabolism of terpenoids and polyketides” (216 genes) can be used to explore the biosynthesis pathways of active ingredients in *M. officinalis*.

**Table 2:** Statistics of predicted protein-coding genes in the *M. officinalis* genome

Gene set	Number	Average gene length (bp)	Average CDS length (bp)	Average exon number per gene	Average exon length (bp)	Average intron length (bp)
De novo	27,698	3217.87	1134.10	5.10	222.27	507.94
Homology	18,549	3740.08	1314.22	5.44	241.60	546.40
RNA-seq	14,338	4787.57	1402.49	5.96	235.14	681.86
Final set	27,102	3762.35	1169.11	5.00	233.60	647.53

We conducted BUSCO analysis to verify the predicted genes. The results showed that approximately 96.80% of the complete BUSCOs could be identified in the annotated results, indicating the high reliability of the predicted results (Supplementary Table S8). The number of predicted genes and structural characteristics of the *M. officinalis* genome were consistent with those of related species, which indicated that the annotation results were acceptable (Supplementary Table S9).

### Gene families and phylogenetic relationships

OrthoMCL software was used to identify the gene families of *M. officinalis* and nine other species, among which *C. canephora* is the most closely related species to *M*. *officinalis* and also belongs to the Rubiaceae family. A total of 22,750 (83.94%) genes were categorized into 14,124 gene families, 849 of which were unique to *M. officinalis* (Supplementary Table S10). In each species, 1576 genes were identified as single-copy orthologs. A total of 7230 genes in *M. officinalis* did not cluster with the genes of other species, indicating that these genes were *M. officinalis* specific (Supplementary Fig. S5a, b). Furthermore, we performed KEGG enrichment analysis to explore the metabolic pathways involved in these species-specific genes. Interestingly, we found that the pathways related to the synthesis of secondary metabolites were significantly enriched (*q* value < 0.05), including “indole alkaloid biosynthesis” (36 genes), “phenylpropanoid biosynthesis” (103 genes), and “stilbenoid, diarylheptanoid, and gingerol biosynthesis” (29 genes) (Supplementary Table S11). Moreover, we also identified 149 genes involved in “plant–pathogen interaction”. These data will help to reveal the molecular mechanisms underlying the interactions between *M. officinalis* and pathogens.

**Fig. 3:**
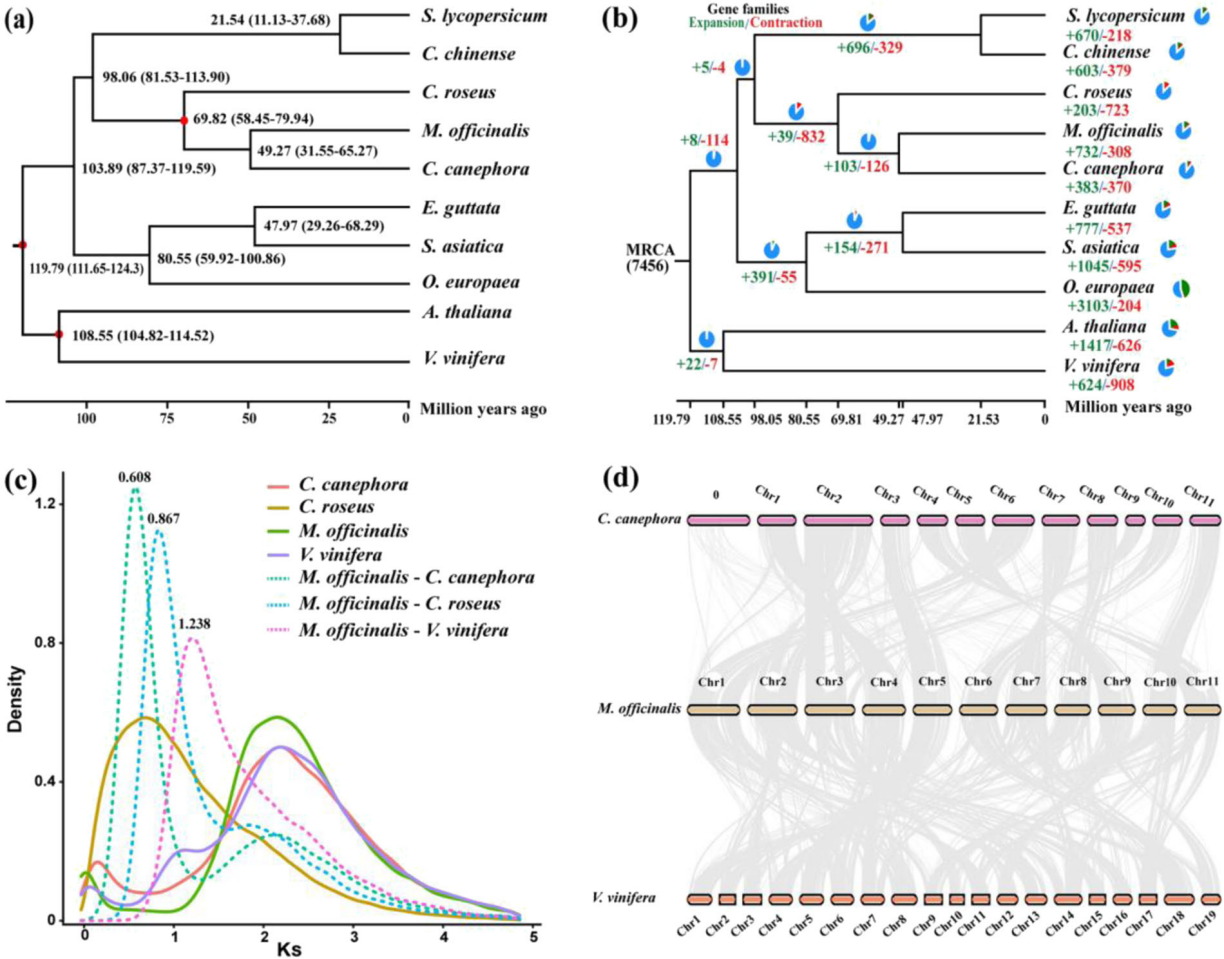
The genome evolution of *M. officinalis*. **a** Phylogenetic tree and divergence time estimation. The value on each node represents the divergence time of each species. The red dots depict the fossil record used to correct the divergence time. **b** Gene family expansion (green) and contraction (red). **c** Ks distribution among *M. officinalis* and three other species. Lines show Ks distribution within (continuous) and between genomes (dashed). **d** Collinear relationship of *M. officinalis*, *C. canephora* and *Vitis vinifera*. The gray line connects matched gene pairs

We used MAFFT software to perform multiple sequence alignments on the identified single-copy orthologous genes. The phylogenetic tree was constructed using the PROTGAMMAAUTO model of RAXML software, in which *V. vinifera* and *Arabidopsis thaliana* were outgroup species. As shown in Fig. 3a, *M. officinalis* was most closely related to *C. canephora*, and the divergence time of the two was 49.27 (31.55–65.27) million years ago (Mya). Comparative genomic analysis showed that 732 expanded and 308 contracted gene families were discovered in *M. officinalis* (Fig. 3b). Compared with the closely related species of *C. canephora* (383 expansion/370 contraction), *M. officinalis* showed more gene family expansion than contraction. KEGG enrichment analysis of the expanded genes suggested that they were mainly enriched in “ABC transporters” (30 genes), “starch and sucrose metabolism” (78 genes), “phenylpropanoid biosynthesis” (97 genes), “isoquinoline alkaloid biosynthesis” (19 genes) and so on, indicating that some of these might be related to the biosynthesis of active compounds (Supplementary Table S12). The contracted gene families were involved in “ether lipid metabolism” (8 genes), “endocytosis” (20 genes), “sesquiterpenoid and triterpenoid biosynthesis” (5 genes), “spliceosome” (18 genes), “protein processing in endoplasmic reticulum” (18 genes) and “plant–pathogen interaction” (25 genes), indicating that some of these families may be related to environmental adaptation (Supplementary Table S13). Notably, we found that a series of genes related to secondary metabolism and environmental adaptation exhibited significant expansion (Supplementary Table S14). These results provide valuable resources for understanding the biosynthesis of active ingredients and the interaction between *M. officinalis* and its growth environment.

### Positive selection, WGD, and collinearity

The *Ka/Ks* ratios of the single-copy genes were used to evaluate the positive selection of genes in *M. officinalis*. A total of 101 candidate genes were strictly positively selected (*p*-value < 0.05) (Supplementary Table S15). GO enrichment analysis showed that these genes were enriched in “DNA repair”, “ATP-dependent helicase activity”, “chromosome”, and “DNA topological change”, indicating that these positively selected genes may improve DNA damage resistance in adverse environments. To estimate the potential WGD events of *M. officinalis*, synonymous nucleotide substitutions (Ks) were characterized in *M. officinalis, C. canephora, Catharanthus roseus,* and *V. vinifera*. Based on the Ks distribution between *M. officinalis* and the other three genomes, *M. officinalis*–*C. canephora* (0.608) divergence time was slower than that of *M. officinalis–C. roseus* (0.867) and *M. officinalis–V. vinifera* (1.238), which was consistent with the phylogenetic tree (Fig. 3c). The distribution of Ks showed one peak at ~2.1 to 2.3 in the genomes of *M. officinalis*, *C. canephora,* and *V. vinifera*. The results showed that they shared an ancient WGD event (the core eudicot γ triplication event) before their divergence and no recent independent WGD event (Fig. 3c). The comparative genome structure between *M. officinalis* and *C. canephora* showed high collinearity, indicating that there was no large-scale structural variation after the divergence between *M. officinalis* and *C. canephora* (Fig. 3d and Supplementary Fig. S6). For most collinear regions, one chromosome of *M. officinalis* corresponded to one chromosome of *C. canephora*; for example, MoChr2, MoChr5, MoChr6, MoChr7, MoChr8, MoChr9, and MoChr10 of *M. officinalis* corresponded to CcChr2, CcChr3, CcChr10, CcChr7, CcChr8, CcChr5, CcChr11 and CcChr4 of *C. canephora*, respectively. MoChr1 corresponded to CcChr1, CcChr2 and CcChr6; MoChr3 corresponded to CcChr2, CcChr6 and CcChr9; MoChr4 corresponded to CcChr1, CcChr3, and CcChr6 (Supplementary Fig. S6). These observations indicated that MoChr1, MoChr3, and MoChr4 might have formed by fragmentation and recombination of ancestral chromosomes. We further conducted intergenomic collinearity between *M. officinalis* and *V. vinifera*. The *M. officinalis* genome generally showed a one-to-one syntenic relationship with *V. vinifera*, which was consistent with the result that the *M. officinalis* genome did not undergo a recent WGD event (Fig. 3d). Interestingly, the collinear regions of MoChr5 mainly corresponded to VvChr5, and MoChr6 corresponded to VvChr 18, while other chromosomes, especially MoChr1, MoChr2, and MoChr3, did not exhibit any significant corresponding relationships between *M. officinalis* and *V. vinifera* (Supplementary Fig. S6).

### Characteristic analysis of genes showing organ-specific expression

**Fig. 4:**
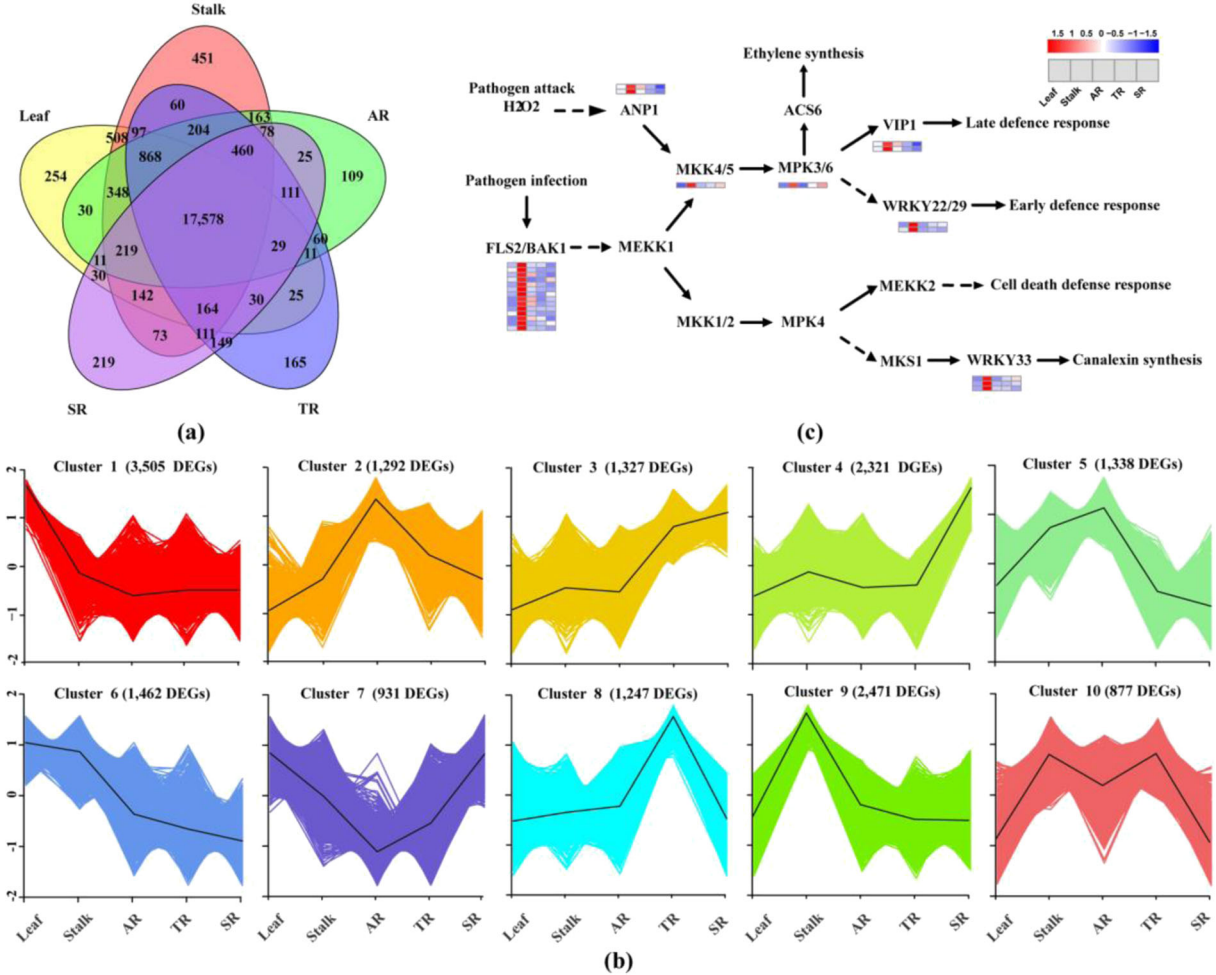
Gene expression patterns in different tissues. **a** Venn diagram showing the expressed genes in roots, stalks, and leaves (FPKM > 0.1). The overlapping regions represent genes expressed in at least two tissues, while the nonoverlapping regions represent tissue-specific genes. **b** Hierarchical clustering showing the expression patterns of DEGs. The *x* axis represents different tissues, and the *y* axis represents the standardized gene expression level based on the log_2_ (FPKM + 1) values. AR, one-year-old roots; TR, three-year-old roots; SR, six-year-old roots. **c** Part of the MAPK signaling pathway. Various color blocks represent the normalized gene expression levels of the DEGs in different tissues

Based on gene expression levels, we identified 451, 254, 109, 165, and 219 genes expressed explicitly in stalks, leaves, one-year-old roots (AR), three-year-old roots (TR), and six-year-old roots (SR), respectively, and 17,578 genes were expressed in all tissues (Fig. 4a). To elucidate the similarities and differences of gene expression patterns in different tissues, we also performed a k-means cluster analysis. A total of 16,771 differentially expressed genes (DEGs) were divided into 10 clusters (Fig. 4b). We, therefore, focused our attention on the clusters that contained genes with tissue-specific expression. We found that the leaf-biased genes (cluster 1) were highly correlated with fundamental pathways, such as “photosynthesis”, “biosynthesis of secondary metabolites”, “photosynthesis-antenna proteins”, “carbon fixation in photosynthetic organisms” and “porphyrin and chlorophyll metabolism” (Supplementary Table S16). In contrast, stalk-biased genes (cluster 9) were significantly associated with defense responses, such as “MAPK signaling pathway-plant” and “plant–pathogen interaction” (Fig. 4c and Supplementary Table S16). Additionally, we also identified that genes in clusters 2, 4, and 8 were highly expressed at different developmental stages of roots, and some genes were continuously enhanced with root development (clusters 3 and 7). The functions of these genes were mainly enriched in “phenylpropanoid biosynthesis”, “amino sugar and nucleotide sugar metabolism”, “ABC transporters”, “MAPK signaling pathway-plant”, “plant–pathogen interaction” and “peroxisome”, indicating that they may be related to the synthesis, transport, and storage of active ingredients and defense responses (Supplementary Table S16). These results provide a basis for further analysis to reveal the gene expression regulatory network and regulate bioactive metabolite derivative production in *M. officinalis*.

### Identification of genes related to the anthraquinone biosynthesis pathway

**Fig. 5:**
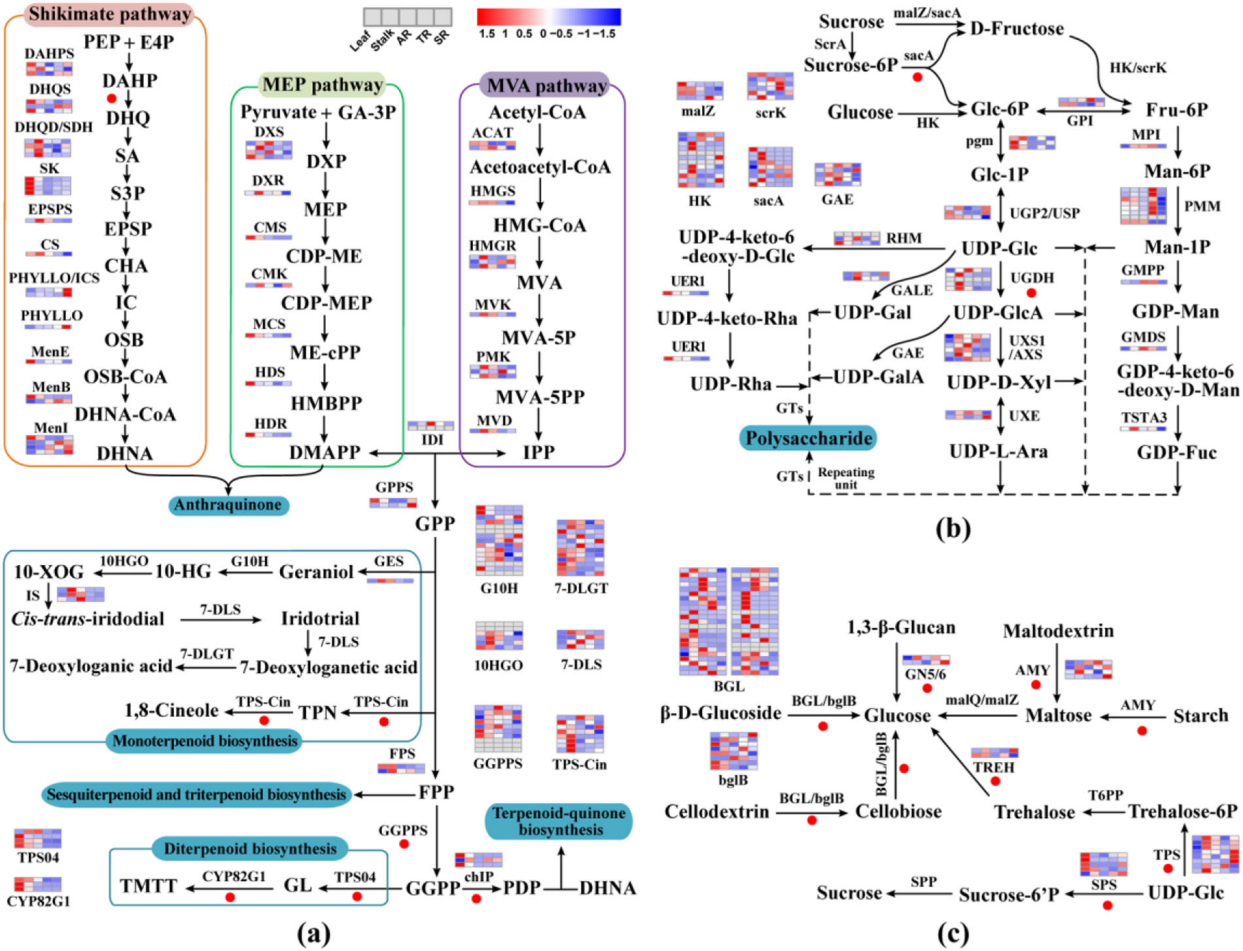
Expression analysis of genes involved in major active compound biosynthesis. **a** Anthraquinone and terpenoid biosynthesis pathways. **b** Polysaccharide biosynthesis pathway. **c** Part of the starch and sucrose metabolism pathway. Various color blocks represent the normalized gene expression levels of all genes encoding in different tissues. Heat maps of these genes were plotted using the pheatmap package. Red dots represent gene family expansion in *M. officinalis*

Anthraquinones in Rubiaceae plants are mainly synthesized through the shikimate/*o*-succinylbenzoic acid pathway, which mainly involves the shikimate pathway, TCA cycle, mevalonate (MVA) pathway, and methylerythritol phosphate (MEP) pathway (Fig. 5a). 1,4-Dihydroxy-2-naphthoate (DHNA) is formed by isochorismate and α-ketoglutarate under the catalysis of a series of enzymes. Therefore, DHNA combines with dimethylallyl pyrophosphate (DMAPP), derived from the MVA and MEP pathways, to form the core structure of anthraquinone. We identified 11 crucial gene families in the shikimate pathway (Supplementary Table S17). In addition, compared with *C. canephora*, the key gene DHQS in the shikimate pathway was expanded to enhance the ability to produce 3-dehydroquinate (DHQ). On the other hand, the chlP gene number was also expanded, which may contribute to terpenoid-quinone biosynthesis. We also identified 14 important gene families in the MVA and MEP pathways (Fig. 5a and Supplementary Table S17). Almost every node in the MEP pathway had only one gene copy, while the crucial genes ACAT, HMGR and PMK in the MVA pathway had two or three gene copies. Gene expression analysis showed that most MEP pathway genes had high expression in leaves, while genes in the MVA pathway were expressed in various tissues.

### Functional gene evolution contributed to the formation of terpenoids

**Fig. 6:**
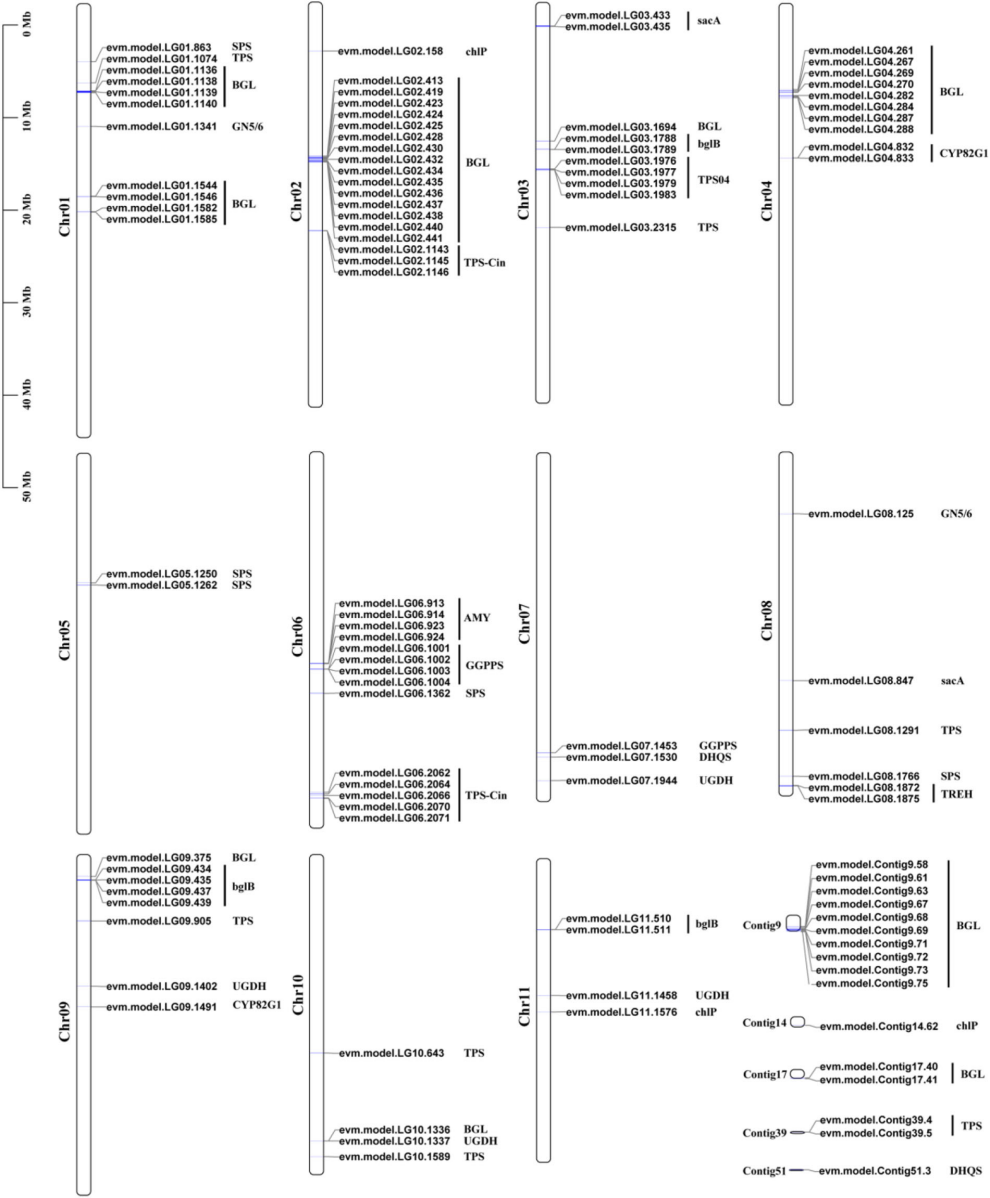
Chromosomal locations of expanded gene families in the M. officinalis genome. Gene families related to anthraquinone and terpenoid biosynthesis pathways (chlP, CYP82G1, DHQS, GGPPS, TPS04 and TPS-Cin) and sugar metabolism (sacA, bglB, AMY, BGL, GN5/6, UGDH, SPS, TPS and TREH)

Terpenoid biosynthesis starts from the terpenoid backbone biosynthesis pathway. Isopentenyl pyrophosphate (IPP) and DMAPP are common precursors for terpenoid biosynthesis in plants and are mainly formed by the MVA and MEP pathways. Geranylgeranyl diphosphate (GGPP) is the direct precursor substrate of diterpene biosynthesis, which GGPPS catalyzes. In the *M. officinalis* genome, we found that GGPPS has ten gene copies, and tandem duplication was found on Chr6 (Fig. 6 and Supplementary Table S17). Gene expression analysis showed that GGPPS might have potential functional divergence; for example, *evm.model.LG06.1002* was relatively highly expressed in AR, but *evm.model.LG06.1003* showed no expression in any tissues (Supplementary Table S17).

Terpene synthase (TPS) is vital for terpenoid synthesis and uses geranyl diphosphate (GPP), GGPP, and farnesyl diphosphate (FPP) as direct precursors to synthesize monoterpenes, diterpenes, sesquiterpenes, and triterpenes. TPS proteins were identified by using the Pfam domain models PF03936 and PF01397 with an *E*-value cutoff of 1e−5. We identified 41 TPS family genes in the *M. officinalis* genome. Based on the phylogenetic tree generated by aligning the TPS proteins of *M. officinalis*, *C. canephora,* and *A. thaliana*, we divided the *M. officinalis* TPS genes into seven groups (Supplementary Fig. S7a and Supplementary Table S18). The TPS-a (14 genes) and TPS-b (14 genes) groups contained more genes, while the TPS-g group comprised only two genes. We also found that the TPS-a group had tandem duplication on Chr2 (10 genes), and the TPS-b group had tandem duplication on Chr2 (6 genes) and Chr6 (6 genes) (Supplementary Table S18). Thus, tandem duplication is responsible for TPS-Cin and TPS04 gene family expansion in *M. officinalis* after its split from *C. canephora* (Fig. 6). In addition, we noticed that the expression patterns of most genes in the same group were similar. Nevertheless, TPS-a was mainly expressed in AR, and TPS-b showed high expression in stalks, which may be related to the tissue-specific localization of substance synthesis (Supplementary Fig. S7b and Supplementary Table S18).

Iridoids are the major terpenoids in *M. officinalis* and are monoterpene analogs. In the iridoid biosynthesis pathway, one GES (TPS-g subfamily), fifteen G10Hs, six 10HGOs, three ISs, four 7-DLSs and twelve 7-DLGTs were identified (Fig. 5a and Supplementary Table S17). Based on the chromosome location, we found that these functional genes (G10H, 10HGO, IS, 7-DLS, and 7-DLGT) may have undergone tandem duplication in the *M. officinalis* genome. Interestingly, this duplication also existed in *C. canephora*, which suggested that the duplication of these gene families may have occurred before the speciation of *M. officinalis* and *C. canephora*.

### Significant expansion of genes related to sugar metabolism

As shown in Fig. 5b, we deduced the synthesis pathway of polysaccharides in *M. officinalis* based on the enzymes involved in carbon metabolism in the pathways “starch and sucrose metabolism” and “amino and nucleotide sugar metabolism”. In *M. officinalis*, nine genes encoding sacA, four genes encoding malZ, six genes encoding scrK, and 12 genes encoding HK were identified, most of which were highly expressed in leaves and stalks (Fig. 5b and Supplementary Table S19). We also identified 44 genes encoding nucleotide-diphospho-sugar interconversion enzymes, most of which showed diverse expression patterns in different tissues, indicating the complexity of the regulation of polysaccharide biosynthesis (Fig. 5b and Supplementary Table S19). The sacA gene catalyzes the formation of D-fructose and Glc-6P as precursors. Two tandem repeat blocks were found on Chr1 and Chr3 (Fig. 6). UGDH is responsible for the biosynthesis of UDP-GlcA, which might be the restrictive precursor of other nucleotide-diphospho-sugars, such as UDP-Gal, UDP-d-Xyl and UDP-l-Ara. We found that the UGDH gene in *M. officinalis* was expanded compared to that in *C. canephora*, which might have contributed to the formation of UDP-GlcA (Fig. 6 and Supplementary Table S20).

We also paid particular attention to other expanded gene families related to the starch and sucrose metabolism pathways (Fig. 5c). Based on their function, these extended genes catalyze the formation of glucose from other sugars, which is one of the important substrates for glycolysis and polysaccharide synthesis. A significant tandem duplication event of BGL genes was identified in *M. officinalis* on Chr1, Chr2, Chr4, contig9, and contig 17 (Fig. 6). For the bglB genes, similar tandem repeats were found on Chr3, Chr9, and Chr11 (Fig. 6 and Supplementary Table S20). The AMY genes can decompose starch into maltose and then form glucose under the catalysis of malQ and malZ. Tandem duplication of AMY genes was also found on Chr6 (Fig. 6 and Supplementary Table S20). These results suggested that tandem duplication is responsible for the expansion of these genes after the separation between *M. officinalis* and *C. canephora*.

## Discussion

*M. officinalis* is one of the top four most famous southern herbs in China. Nevertheless, in the past, research on *M. officinalis* has mainly focused on its pharmacology. Because of the impediments associated with the sexual reproduction of this species and the exhaustion of its wild resources, germplasm resources are very limited^15^. Additionally, the lack of genomic information seriously hinders genetic research on *M. officinalis*. Therefore, obtaining genomic information on *M. officinalis* will enable potential genetic improvements and the development of molecular breeding resources. However, a genome survey showed that its genome was highly heterozygous (Supplementary Fig. S1). High repetition rates and high heterozygosity are challenges for high-quality genome assembly^23^. To overcome this issue, we combined third-generation sequencing and Hi-C technology to assemble the *M. officinalis* genome. Here, we present a high-quality chromosome-scale genome sequence for *M. officinalis*, with a scaffold N50 of 40.97 Mb, which is higher than that of other medicinal plants, such as *S. baicalensis* (408.14 Mb, N50 33.2 Mb)^19^. A total of 97.02% complete BUSCO core genes were detected in our assembly, which suggested that the quality of this reference genome is comparable to that of the published highly heterozygous *T. wilfordii* genome (95.10%)^24^. In brief, the assembly of *M. officinalis* is relatively accurate and complete, which will provide a valuable genome resource for understanding the evolution, active ingredient biosynthesis, and genetic improvement of this species.

The genus *Morinda* (Rubiaceae), which includes 102 species, is distributed in tropical, subtropical, and temperate regions of the world^2^. However, there are few reports on genome research of the species in this genus. We found that 82.77% of annotated genes were matched to the proteins of the genus *Coffea* (Rubiaceae). This indicates the lack of reference genome sequences and limited public data on the genus *Morinda*. As one of the members of this genus, the genome information of *M. officinalis* can fill this gap and promote evolutionary research. Ks analysis found that *M. officinalis* shared only an ancient WGD event with *C. canephora* and *V. vinifera*, and no recent WGD event occurred (Fig. 3c). The *M. officinalis* chromosomal regions showed a one-to-one correspondence with *V. vinifera*; similar results were also found between *C. canephora* and *V. vinifera*^25^. WGD events can cause plant genome size variation, chromosomal rearrangement, gene family expansion and species evolution^26,27^. We found a high collinearity relationship between *M. officinalis* and *C. canephora* chromosomes, which may be because they have not undergone recent WGD events or large-scale chromosomal variation after species divergence (Supplementary Fig. S6).

*M. officinalis* diseases are one of the critical factors that affect yield and quality. Stem rot is a common destructive disease of *M. officinalis*, often occurring at the stem base^17^. We identified 59 expanded and 25 contracted genes associated with plant–pathogen interactions in the *M. officinalis* genome (Supplementary Table S14 and Supplementary Fig. S8). These extended genes are located on all chromosomes of *M. officinalis*, and the KCS gene (K15397) and CML gene (K13448) have undergone tandem duplication on Chr5 and Chr6, respectively (Supplementary Fig. S8). This may be a unique adaptative mechanism evolved by *M. officinalis* in response to changes in various pathogens during natural selection. Based on the gene expression patterns, we found increased expression of 2,741 DEGs in the stalks, and functional enrichment showed that these genes were significantly associated with defense responses, such as “MAPK signaling pathway-plant” and “plant–pathogen interaction” (Fig. 4b and Supplementary Table S16). These genes (e.g., FLS2, MPK3/6, and WRKY22) are involved in signal perception, cascade amplification, transmission, and regulation of downstream functional gene expression, which plays a crucial role in plant disease resistance by regulating multiple defenses^28–30^. Therefore, our results provide insights into the molecular mechanism of the interaction between *M. officinalis* and pathogens.

As medicinal and edible plants, *M. officinalis* contains medicinally active compounds, mainly including anthraquinones, iridoids, and polysaccharides^1,2,10^. Although genes involved in anthraquinone, iridoid, and polysaccharide biosynthesis have been identified in other medicinal plants, such as *Ophiorrhiza pumila*, *Senna tora*, *Gardenia jasminoides*, and *Artemisia sphaerocephala*, the biosynthesis pathways in *M. officinalis* are still unclear^31–34^. Previous studies have shown that there are two metabolic pathways in plant anthraquinone biosynthesis. The shikimate/o-succinylbenzoic acid pathway mainly exists in Rubiaceae plants, while the polyketide pathway mainly exists in fungi and other plants, such as Leguminosae, Rhamnaceae, and Polygonaceae^35,36^. Terpenoids are the main active ingredients of many medicinal plants, such as *Andrographis paniculata*, *Gynostemma pentaphyllum* and *Gardenia jasminoides*^37–39^. The synthesis of terpenoids starts from the common precursors IPP and DMAPP, and then TPS converts the corresponding substrates to form structurally diverse monoterpenes, diterpenes, sesquiterpenes, etc.^40^. Polysaccharides are important bioactive components with various activities. Previous studies have shown that the polysaccharides in *M. officinalis* are mainly composed of glucose and fructose^10,11^. In this work, we identified candidate genes related to the biosynthesis of these active components in *M. officinalis* and analyzed their expression patterns in different tissues (Fig. 5a, b). We also found that some essential genes involved in the synthesis of these active ingredients, such as DHQS, GGPPS, TPS-Clin, TPS04, sacA, and UGDH, expanded in *M. officinalis* (Fig. 6). The expansion of key genes in metabolic pathways is beneficial to the synthesis and accumulation of active components and is a common event in medicinal plants during the evolutionary process^41^. Notably, we found that the expression patterns of some expanded genes with the same function were diverse, indicating a potential functional divergence of these gene families.

The roots of *M. officinalis* serve as an effective agent in traditional Chinese medicine, and there are two main stages in plant growth and development in this species: the growth of aboveground tissues and the growth of roots. Therefore, the vines need to be manually cut to promote the expansion of the roots after three years of cultivation, but this process may affect photosynthesis. Glucose is an important substrate for glycolysis and the TCA cycle, which provides energy for biological activities and intermediates for other metabolic processes^42^. Interestingly, we found that gene families involved in converting other sugars to glucose, such as BGL, AMY, and TREH, expanded significantly (Fig. 6). We speculate that these evolutionary genes in *M. officinalis* may need to compensate for glucose deficiency. Although we have identified related candidate genes, their functions and evolutionary mechanisms should be explored in future work.

## Conclusions

In this work, we first report the high-quality chromosome-scale reference genome of *M. officinalis*. Genome evolution showed that *M. officinalis* shared an ancient WGD event with *C. canephora* and *V. vinifera*. We further used high-quality genome information to identify candidate genes for terpenoid, anthraquinone, and polysaccharide biosynthesis. We found that the functional genes related to pathogen resistance and active component biosynthesis were expanded in the *M. officinalis* genome. Overall, this high-quality reference genome provides insights into genome evolution and active component biosynthesis in *M. officinalis*. Our research also lays the foundation for further studies for genetic improvement and breeding, not only in *M. officinalis* but also in other *Morinda* species.

## Materials and methods

### Plant materials and sequencing

Root, leaf, and stem samples of “Gaoji 3”, a cultivated variety of *M. officinalis*, were collected from the Gaoyao District (Zhaoqing city, 28°28′–28°66′ N, 85°13′–85°28′ E) in Guangdong Province, China. Because of its unique geography and environment, the Gaoyao District of Zhaoqing City is considered the authentic production area of *M. officinalis*. “Gaoji 3”, with many excellent cultivation characteristics, including high yield, high quality, and disease resistance, is the main variety of *M. officinalis* grown in Gaoyao District.

High-quality genomic DNA was extracted using a QIAGEN^®^ Genomic Kit (QIAGEN, Germany). The integrity of the DNA was checked by 0.75% agarose gel electrophoresis. The purity and concentration of the DNA were analyzed by using a NanoDrop 2000 spectrophotometer (ThermoFisher Scientific, USA) and Qubit Fluorometer (ThermoFisher Scientific, USA). A paired-end library was constructed and sequenced using the MGISEQ-2000 (BGI, Shenzhen, China) and Illumina NovaSeq 6000 platforms (Illumina, San Diego, USA) to generate short sequencing reads. We constructed DNA libraries for long-read single-molecule sequencing and sequenced them on the Nanopore PromethION platform. Total RNA was extracted from the roots, stems, and leaves using an RNAprep Pure Plant Kit (TIANGEN Biotech, China). The RNA libraries were prepared using a TruSeq RNA Library Kit (Illumina, CA, USA) and then sequenced on the Illumina NovaSeq 6000 platform.

The quality of the short sequencing reads was estimated using the FastQC tool, and the adapter sequences, contamination, PCR duplicates, and low-quality reads (reads with more than 30 low-quality bases or 5% unknown bases) were removed using fastp^43,44^. For the Nanopore data, long raw reads were converted into fastq format after base calling by using the Guppy tool^45^. Reads with a mean_qscore_template value greater than seven were retained^46^. NextDenovo (https://github.com/Nextomics/NextDenovo), with the parameters read_cutoff = 1 kb and seed-cutoff = 37 kb, was employed to ensure further correction and assembly. These clean data were used for further assembly and subsequent analysis.

### Genome survey

To estimate the *M. officinalis* genome characteristics, we used a K-mer-based method to estimate the genome size and heterozygosity. Approximately 61.4 Gb (~127×) clean short reads were generated and used for 17 K-mer analyses (Supplementary Table 1). The frequency distribution of 17 K-mers was counted by Jellyfish software^47^. Then, the K-mer depth distribution curve was calculated to estimate the genome size. To further estimate the heterozygosity rate, the genome of *Arabidopsis thaliana* was used to simulate the corresponding depth of short-read data, and K-mer curve fitting was carried out under different gradient heterozygosity rates. The heterozygosity rate was estimated according to K-mer curve fitting.

### Genome and chromosome assembly

After quality control, a total of 62.92 Gb (~130×) corrected Nanopore long reads were generated with an average length of 22.39 Kb and N50 of 30.16 Kb. These qualified long reads were assembled by using NextGraph with default parameters to obtain the preliminary assembled genome sequence. The Nanopore long reads were mapped to the preliminary assembled genome using minimap2 with the parameters “-x map-ont” and subjected to three rounds of polishing using Nextpolish with default parameters^48,49^. Furthermore, to improve accuracy, the clean paired-end reads were mapped to the genome sequence using bwa to polish the assembled contigs with Pilon (iterative correction of four times)^50,51^. Finally, Redundant software was employed to resolve redundancy in the assembly with the parameters “–identity 0.8–overlap 0.8” to obtain the final nonredundant genome^52^.

To further anchor the contigs to chromosomes, we used fresh young leaves to construct a Hi-C library using a NEBNext Ultra II DNA Library Prep Kit. Approximately 55.8 Gb of clean data were generated for Hi-C analyses by the Illumina NovaSeq 6000 platform. These clean reads were mapped to the *M. officinalis* genome using Bowtie2 software (v2.3.2) with the parameters “–very-sensitive -L 30” to obtain the uniquely mapped paired-end reads^53^. After filtering out the invalid interaction pairs, including dangling end paired-end reads, self-circle paired-end reads, and dumped paired-end reads, valid paired-end reads were identified and separated by using HiC-Pro (v2.7.8)^54^. We then used LACHESIS software to cluster and reorder the contigs into pseudochromosomes with the following parameters: cluster min re sites = 100, cluster max link density = 2.5, cluster noninformative ratio = 1.4, order min n res in trunk = 60, and order min n res in shreds = 60^55^. Finally, the order and direction of the contigs on the pseudochromosomes were evaluated and adjusted by examining their interactions in the Hi-C heatmap.

### Evaluation of the assembled genome

We used multiple methods to assess the accuracy and completeness of the assembled genome. First, the paired-end reads were mapped to the genome to evaluate its completeness using bwa with the default parameters. RNA-seq data from different tissues (leaf, stalk, and root) were also aligned to the reference genome to obtain the mapping rate using HISAT2 with the default settings^56^. Second, GC depth scatter plots were used to evaluate any contamination in the sequencing data. Finally, the accuracy and completeness of the genome assembly were evaluated by using BUSCOs to identify the single-copy genes in the assembled genome with the Embryophyta_odb10 database^57^.

### Repeat element identification

The repetitive sequences in the genome can be divided into two main categories: tandem repeats and transposable elements. We used two software programs, GMATA and Tandem Repeats Finder, to search for tandem repeats in the whole genome with default parameters^58,59^. Homology alignment and de novo searches were combined to identify transposable elements. RepeatModeler was used for de novo searching for repetitive sequences, which were then classified with Teclass^60,61^. We identified the repeats through a homology-based repeat search using Repbase^62^. We also used MITE-hunter to discover the small transposon called MITE^63^. LTR_finder and LTR_harvest software were employed to identify the LTRs, and LTR_retriever was used to integrate these results to obtain an LTR retrotransposon library of *M. officinalis*^64–66^.

### Noncoding RNA prediction

The tRNA genes were predicted using tRNAscan-SE, and other noncoding RNAs, including rRNA, snRNA, and miRNA, were predicted by comparison with the Rfam database using Infernal software with the default parameters^67–69^. At the same time, we also used RNAmmer software to construct models to predict rRNA and its various subunits^70^.

### Gene prediction and functional annotation

Transcriptome-based, homology-based, and ab initio prediction methods were combined to predict gene models in the *M. officinalis* genome. To improve gene prediction, RNA libraries were prepared from mixed fresh leaf, stem, and root tissues, and finally 33.43 Gb clean data were generated. For homology-based annotation, the protein sequences of *C. canephora*^25^, *C. Arabica*^71^, *C. roseus*^72^, and *A. thaliana*^73^ were downloaded and aligned against the *M. officinalis* genome using GeMoMa^74^. For transcriptome-based prediction, the non-redundant transcripts were aligned to the reference genome to obtain gene structures using PASA^75^. Then, TransDecoder was used to search the longest open reading frames according to the PASA results^76^. We chose 3000 genes with the highest alignment scores (identity >95%) as the training sets for the AUGUSTUS model to generate a generalized hidden Markov probability model for ab initio gene prediction^77^. Finally, we integrated the gene models from the three approaches with EvidenceModeler, and TransposonPSI was used to remove genes containing transposable elements to generate the final consensus gene models^78,79^.

Functional annotation of the protein-coding genes was carried out by using Blastp with a cutoff E-value of 1e−5 with different public databases. The functions of the genes were predicted and classified using the KOG, NR, and UniProtKB/SwissProt databases. The GO database classified and annotated genes according to three categories: biological processes, cellular components, and molecular functions. We used InterProScan software to identify protein domains by matching them against Pfam database entries to obtain GO terms^80^. Pathway annotation was performed with the KEGG database with an *E*-value ≤ 1e−5.

### Gene families and phylogenetic analysis

We collected the protein sequences of *M. officinalis* and nine other species, including *A. thaliana*, *Capsicum chinense*, *C. roseus*, *C. canephora*, *Erythranthe guttata*, *Olea europaea*, *Solanum lycopersicum*, *Striga asiatica,* and *V. vinifera*, for evolutionary analysis. An all-to-all BLASTP analysis of the protein sequences of all species was performed with an *E*-value ≤ 1e−5. Subsequently, orthologous genes, paralogous genes, and single-copy homologous genes were identified using OrthoMCL software with the default parameters^81^.

Phylogenetic construction was performed based on the single-copy orthologous genes from the ten species. We used MAFFT software to align the protein sequences and extracted the conserved sites from the alignments using Gblocks^82,83^. We used *V. vinifera* and *A. thaliana* as an outgroup and performed 1000 bootstrap replicates to construct a phylogenetic tree by RaxML^84^. The divergence time of each species was estimated based on the Bayesian relaxed molecular clock approach using MCMCTREE of the PAML package^85^. According to the results of the gene families and phylogenetic tree, λ values were estimated with CAFE software to predict the expansion or contraction of gene families of different species in each evolutionary branch based on a stochastic birth and death process model^86^. Gene families with a *p*-value smaller than 0.05 were considered significantly expanded or contracted.

### Positive selection genes and WGD analysis

Based on the results of single-copy genes, Codeml software was used to calculate the selection pressure to identify the positively selected genes of *M. officinalis* (*p*-value ≤ 0.05)^87^. We performed multiple sequence alignment to extract the conserved paralogs of the protein sequences of *M. officinalis* by using BLASTP (*E*-value ≤ 1e−5). MCScan was used to identify collinearity blocks, and Ks values were calculated by PAML to predict WGD events^88^.

### Transcriptome profiling analysis

A total of 108.94 Gb clean reads were produced from the leaves, stalks, and roots at different developmental stages of *M. officinalis*. These reads were aligned to the reference genome by using HISAT2^56^. The fragments per kilobase of transcript per million fragments mapped (FPKM) were calculated to estimate the expression level of genes. EdgeR was used to analyze the significantly DEGs with an FDR ≤ 0.05 and an absolute log_2_ (fold change) ≥1 as the threshold^89^.

## Supplementary information

Supplementary data

## Data Availability

The sequencing data, including the MGI genome data (SRA accession: SAMN14599083) and Hi-C data (SRA accession: SAMN14599083), are available in the NCBI Sequence Read Archive (SRA) database under BioProject accession number PRJNA625354.
