# Supplementary material for: A high-quality genome assembly of Morinda officinalis, a famous native southern herb in the Lingnan region of southern China
Source: Hortic Res. 2021 Jun 1;8:135. doi: 10.1038/s41438-021-00551-w (PMC8166937; doi:10.1038/s41438-021-00551-w)
Supplement: Supplementary file 1 — Supplementary data [file 41438_2021_551_MOESM1_ESM.pdf]

Supplementary Table S1-S20

Supplementary Figure S1-S8

A high-quality genome assembly of *Morinda officinalis*, a famous native southern herb in the Lingnan region of southern China

Jihua Wang[1#], Shiqiang Xu[1#], Yu Mei[1], Shike Cai[1], Yan Gu[1], Minyang Sun[1], Zhan Liang[4], Yong Xiao[3]*, Muqing Zhang[2]* and Shaohai Yang[1]*

Supplementary Table S1. Sequencing data used for *M. officinalis* genome assembly

Types	Sequencing platform	Clean data (Gb)	Sequence coverage (X)[a]	Use of the data
Genome	MGISEQ-2000	61.4	127	Genome estimation and polishing
Genome	Nanopore PromethION	62.92	130	Genome assembly
Hi-C	Illumina novaseq 6000	55.8	115	Chromosome construction
Transcriptome	Illumina novaseq 6000	33.43	-	Gene annotation
Transcriptome	Illumina novaseq 6000	108.94	-	Gene expression analysis

[a]The sequence coverage was calculated based on the estimated genome size of k-mer analysis.

Supplementary Table S2. Statistics of the pseudochromosome length by Hi-C assisted assembly

Pseudochromosome	Length (bp)	Contig number
Chr01	46,998,976	18
Chr02	43,901,840	18
Chr03	43,568,606	15
Chr04	43,532,616	26
Chr05	41,262,427	25
Chr06	40,972,926	17
Chr07	37,855,901	12
Chr08	37,275,217	16
Chr09	36,946,065	16
Chr10	34,709,186	12
Chr11	33,060,658	9
Total	440,084,418	184

Supplementary Table S3. Statistics of BUSCO estimation for *M. officinalis* genome assembly

Types	Number	Percentage (%)
Complete BUSCOs (C)	1,334	97.02
Complete and single-copy BUSCOs (S)	1,241	90.25
Complete and duplicated BUSCOs (D)	93	6.76
Fragmented BUSCOs (F)	14	1.02
Missing BUSCOs (M)	27	1.96
Total BUSCO groups searched	1,375	--

Supplementary Table S4. Statistics the rate of short reads mapped to the assembled genome

Total reads	Map reads	Map rate	Paired reads	Paired map reads	Properly paired reads	Properly map rate
404,484,756	401,483,949	99.26%	401,084,490	397,529,538	380,889,964	94.97%

Supplementary Table S5. Percentages of RNA-seq reads mapped to the reference genome

Samples	Total reads	Reads mapped	Unique mapped	Multi mapped	'+' mapped	'-' mapped
Leaf1	47,296,676	44,858,882 (94.85%)	41,755,907 (88.29%)	4,476,596 (6.56%)	20,854,649 (44.09%)	20,901,258 (44.19%)
Leaf2	49,741,502	47,253,151 (95.00%)	43,936,735 (88.33%)	4,776,972 (6.67%)	21,944,760 (44.12%)	21,991,975 (44.21%)
Leaf3	50,603,596	47,999,572 (94.85%)	44,740,788 (88.41%)	4,553,765 (6.44%)	22,345,214 (44.16%)	22,395,574 (44.26%)
Stalk1	49,199,200	46,190,343 (93.88%)	43,476,470 (88.37%)	3,762,070 (5.52%)	21,719,112 (44.15%)	21,757,358 (44.22%)
Stalk2	49,100,994	46,039,000 (93.76%)	43,369,143 (88.33%)	3,696,497 (5.44%)	21,659,168 (44.11%)	21,709,975 (44.21%)
Stalk3	50,211,534	47,176,038 (93.95%)	444,189,98 (88.46%)	3,817,444 (5.49%)	22,187,246 (44.19%)	22,231,752 (44.28%)
AR1	46,670,626	43,874,718 (94.01%)	41,168,403 (88.21%)	3,903,106 (5.80%)	20,557,015 (44.05%)	20,611,388 (44.16%)
AR2	49,508,634	46,449761 (93.82%)	43,538,066 (87.94%)	4,257,405 (5.88%)	21,736,132 (43.90%)	21,801,934 (44.04%)
AR3	41,812,910	39,278,019 (93.94%)	36,845,604 (88.12%)	3,488,899 (5.82%)	18,394,634 (43.99%)	18,450,970 (44.13%)
TR1	47,987,392	45,261,832 (94.32%)	42,411,236 (88.38%)	4,103,829 (5.94%)	21,185,196 (44.15%)	21,226,040 (44.23%)
TR2	49,969,76	47,273,037 (94.60%)	44,350,112 (88.76%)	4,156,261 (5.85%)	22,151,193 (44.33%)	22,198,919 (44.43%)
TR3	48,490,400	45,964,341 (94.79%)	43,096,745 (88.88%)	4,133,905 (5.91%)	21,531,718 (44.40%)	21,565,027 (44.47%)
SR1	48,482,100	46,449,144 (95.81%)	43,867,607 (90.48%)	3,544,124 (5.32%)	21,903,590 (45.18%)	21,964,017 (45.30%)
SR2	45,555,10	43,725,193 (95.98%)	41,354,120 (90.78%)	3,227,767 (5.20%)	20,652,688 (45.34%)	20,701,432 (45.44%)
SR3	51,689,674	49,662,698 (96.08%)	46,957,715 (90.85%)	3,678,772 (5.23%)	23,449,799 (45.37%)	23,507,916 (45.48%)

Supplementary Table S6. Statistics of repeat elements of *M. officinalis* assembly

Types	Number	Length of sequence (bp)	Percentage of sequence (%)
Class I	529,580	208,092,431	42.92
Class I/LTR	430,725	173,520,970	35.79
Class I/LTR/Unknown	286,415	88,929,208	18.34
Class I/LTR/Copia	49,260	22,273,506	4.59
Class I/LTR/Gypsy	91,422	60,344,381	12.45
Class I/LTR/Caulimovirus	3,622	1,973,519	0.41
Class I/LTR/Other	6	356	0
Class I/SINE	10,597	772,449	0.16
Class I/SINE/Unknown	10,307	763,592	0.16
Class I/SINE/Other	290	8,857	0
Class I/LINE	88,258	33,799,012	6.97
Class I/LINE/Unknown	60,439	13,087,056	2.7
Class I/LINE/L1	20,060	17,750,597	3.66
Class I/LINE/RTE-BovB	7,424	2,782,830	0.57
Class I/LINE/Other	335	178,529	0.04
Class II	216,898	54,617,985	11.26
Class II/DNA	171,050	42,853,221	8.84
Class II/DNA/Unknown	134,189	25,837,584	5.33
Class II/DNA/MULE-MuDR	18,001	9,657,439	1.99
Class II/DNA/CMC-EnSpm	8,268	2,785,263	0.57
Class II/DNA/PIF-Harbinger	4,792	2,167,463	0.45
Class II/DNA/hAT-Ac	2,709	1,164,441	0.24
Class II/DNA/hAT-Tag1	1,450	718,553	0.15
Class II/DNA/hAT-Tip100	1,023	485,132	0.1
Class II/DNA/Other	618	37,346	0.01
Class II/MITE	38,661	7,443,772	1.54
Class II/RC	7,187	4,320,992	0.89
Class II/RC/Helitron	7,159	4,320,140	0.89
Class II/RC/Other	28	852	0
SSR	32,225	442,199	0.09
Tandem_repeat	34,896	3,191,340	0.66
Unknown	86,605	14,781,346	3.05
Simple repeats	883	70,462	0.01
Other	1,568	214,901	0.04
Low complexity	22	2,226	0
Total repeats	902,677	281,412,890	58.04

Supplementary Table S7. Statistics of non-coding RNA prediction in *M. officinalis* genome

	Types	Number	Average length (bp)	Total length (bp)	Percentage
rRNA	18S	17	1,967.00	33,439	0.0069%
	28S	18	4,403.33	79,260	0.0163%
	5.8S	3	158.33	475	0.0001%
	5S	171	113.32	19,377	0.0040%
Small RNA	snRNA	207	95.99	19,869	0.0041%
	miRNA	78	141.24	11,017	0.0023%
	spliceosomal	76	140.09	10,647	0.0022%
	other	1,079	110.80	119,551	0.0247%
Regulatory	cis-regulatory elements	5	65.20	326	0.0001%
Regulatory	tRNA	644	75.61	48,690	0.0100%

Supplementary Table S8. Evaluation of the genome annotation by BUSCO analysis

Types	Number	Percentage (%)
Complete BUSCOs (C)	1,331	96.80
Complete and single-copy BUSCOs (S)	1,244	90.47
Complete and duplicated BUSCOs (D)	87	6.33
Fragmented BUSCOs (F)	18	1.31
Missing BUSCOs (M)	26	1.89
Total BUSCO groups searched	1,375	100.00

Supplementary Table S9. Comparison of *M. officinalis* and other related species genome

Species	Gene number	Average transcript length (bp)	Average CDS length (bp)	Average exon number per gene	Average exon length (bp)	Average intron length (bp)
M. officinalis	27,102	3,762.35	1,169.11	5.0	233.6	647.53
C. canephora	25,574	3,188.4	1,205.55	5.1	236.22	483.2
C. roseus	34,363	3,338.54	1,065.13	5.12	208.11	552.06
C. arabica	44,674	3,577.82	1,370.5	4.99	274.51	552.87
A. thaliana	27,444	1,857.35	1,205.78	5.09	236.78	159.22

Supplementary Table S10. Statistics of the number of gene families in *M. officinalis* and nine other species

Species	Genes number	Genes number in families	Family number	Unique families number	Average genes number per family
A. thaliana	27,412	23,046	12,640	843	1.82
C. canephora	25,574	20,875	13,824	466	1.51
C. chinense	34,974	27,096	14,212	885	1.91
C. roseus	34,363	29,212	13,239	675	2.21
E. guttata	27,027	24,297	13,511	402	1.8
M. officinalis	27,102	22,750	14,124	849	1.61
O. europaea	39,631	32,988	14,029	746	2.35
S. asiatica	33,209	23,380	13,144	1,203	1.78
S. lycopersicum	25,355	23,222	14,381	205	1.61
V. vinifera	25,382	22,981	13,478	406	1.71

Supplementary Table S11. KEGG enrichment analysis of the *M. officinalis*-specific genes

KEGG pathway	Ko ID	Frequency	*p*-value	Corrected *p*-value	Count
Plant-pathogen interaction	ko04626	149/1388	2.64E-11	3.23E-09	149
Indole alkaloid biosynthesis	ko00901	36/1388	1.12E-10	6.85E-09	36
Glutathione metabolism	ko00480	43/1388	3.76E-05	1.53E-03	43
Stilbenoid, diarylheptanoid and gingerol biosynthesis	ko00945	29/1388	6.58E-05	2.01E-03	29
Phenylpropanoid biosynthesis	ko00940	103/1388	5.78E-04	1.33E-02	103
Spliceosome	ko03040	73/1388	6.56E-04	1.33E-02	73

Supplementary Table S12. KEGG enrichment analysis of the expanded genes in *M. officinalis*

KEGG pathway	Ko ID	Frequency	*p*-value	Corrected *p*-value	Count
Cyanoamino acid metabolism	ko00460	60/823	3.98E-20	4.26E-18	60
ABC transporters	ko02010	30/823	2.32E-17	1.24E-15	30
Starch and sucrose metabolism	ko00500	78/823	2.46E-14	7.52E-13	78
Phenylpropanoid biosynthesis	ko00940	97/823	2.81E-14	7.52E-13	97
Phenylalanine metabolism	ko00360	42/823	2.33E-13	4.98E-12	42
Isoquinoline alkaloid biosynthesis	ko00950	19/823	9.14E-09	1.63E-07	19
Taurine and hypotaurine metabolism	ko00430	17/823	1.38E-08	2.11E-07	17
Fatty acid elongation	ko00062	19/823	1.27E-06	1.70E-05	19
Linoleic acid metabolism	ko00591	11/823	1.45E-05	1.72E-04	11
beta-Alanine metabolism	ko00410	22/823	2.97E-05	3.18E-04	22
Ribosome biogenesis in eukaryotes	ko03008	30/823	4.56E-05	4.44E-04	30
Betalain biosynthesis	ko00965	9/823	5.21E-05	4.52E-04	9
Tyrosine metabolism	ko00350	21/823	5.49E-05	4.52E-04	21
Fatty acid biosynthesis	ko00061	17/823	3.42E-04	2.62E-03	17
Butanoate metabolism	ko00650	11/823	1.05E-03	7.49E-03	11
Circadian rhythm - plant	ko04712	15/823	1.14E-03	7.65E-03	15
Propanoate metabolism	ko00640	14/823	1.57E-03	9.88E-03	14

Supplementary Table S13. KEGG enrichment analysis of the contracted genes in *M. officinalis*

KEGG pathway	Ko ID	Frequency	*p*-value	Corrected *p*-value	Count
Ether lipid metabolism	ko00565	8/208	2.26E-06	1.07E-04	8
Endocytosis	ko04144	20/208	2.90E-06	1.07E-04	20
Sesquiterpenoid and triterpenoid biosynthesis	ko00909	5/208	6.86E-04	1.41E-02	5
Spliceosome	ko03040	18/208	7.65E-04	1.41E-02	18
Protein processing in endoplasmic reticulum	ko04141	18/208	2.08E-03	3.08E-02	18
Plant-pathogen interaction	ko04626	25/208	3.10E-03	3.83E-02	25

Supplementary Table S14. Statistics of the expanded and contracted genes related to secondary metabolism and environmental adaptation

KEGG B class	Pathway	Gene number	
		Expansion	Contraction
Biosynthesis of other secondary metabolites	Phenylpropanoid biosynthesis	97	9
Biosynthesis of other secondary metabolites	Flavonoid biosynthesis	17	0
Biosynthesis of other secondary metabolites	Stilbenoid, diarylheptanoid and gingerol biosynthesis	14	0
Biosynthesis of other secondary metabolites	Tropane, piperidine and pyridine alkaloid biosynthesis	10	2
Biosynthesis of other secondary metabolites	Isoquinoline alkaloid biosynthesis	19	2
Biosynthesis of other secondary metabolites	Monobactam biosynthesis	2	0
Biosynthesis of other secondary metabolites	Betalain biosynthesis	9	0
Biosynthesis of other secondary metabolites	Indole alkaloid biosynthesis	13	0
Environmental adaptation	Plant-pathogen interaction	59	25
Environmental adaptation	Circadian rhythm - plant	15	1

Supplementary Table S15. Positively selected genes in *M. officinalis*

Group	*p* -value	Postive site number	Gene ID	Swissprot function
ortholog11547	0.0487748	2	evm.model.LG02.1794	Beclin-1-like protein OS=Arabidopsis thaliana OX=3702 GN=ATG6 PE=1 SV=2
ortholog19405	0.0016085	1	evm.model.LG04.1226	Nodulin homeobox OS=Arabidopsis thaliana OX=3702 GN=NDX PE=2 SV=1
ortholog09209	0.0030007	1	evm.model.LG06.989	Putative pentatricopeptide repeat-containing protein At1g19290 OS=Arabidopsis thaliana OX=3702 GN=At1g19290 PE=3 SV=2
ortholog05504	0.0023047	2	evm.model.LG08.887	Deoxyribodipyrimidine photo-lyase OS=Arabidopsis thaliana OX=3702 GN=PHR1 PE=2 SV=1
ortholog16257	0.0269896	1	evm.model.LG08.1059	Beta-adaptin-like protein C OS=Arabidopsis thaliana OX=3702 GN=BETAC-AD PE=1 SV=2
ortholog03784	0.0051927	4	evm.model.LG04.1167	General transcription and DNA repair factor IIH helicase subunit XPD OS=Arabidopsis thaliana OX=3702 GN=XPD PE=1 SV=1
ortholog14590	0.0407451	1	evm.model.LG04.238	
ortholog16385	0.0308672	4	evm.model.LG08.1763	MATH domain and coiled-coil domain-containing protein At3g58210 OS=Arabidopsis thaliana OX=3702 GN=At3g58210 PE=4
ortholog06082	8.21E-05	26	evm.model.LG10.579	Protein OBERON 3 OS=Arabidopsis thaliana OX=3702 GN=OBE3 PE=1 SV=1
ortholog20661	1.58E-05	3	evm.model.LG04.106	GTPase Der OS=Synechococcus sp. (strain CC9605) OX=110662 GN=der PE=3 SV=1
ortholog06212	0.0106479	1	evm.model.LG11.1423	Replication protein A 70 kDa DNA-binding subunit B OS=Arabidopsis thaliana OX=3702 GN=RPA1B PE=3 SV=1
ortholog16812	0.0240242	1	evm.model.LG03.51	Zinc finger-containing ubiquitin peptidase 1 OS=Mus musculus OX=10090 GN=Zup1 PE=2 SV=2
ortholog12083	0.0053212	3	evm.model.LG02.2302	
ortholog11321	7.34E-05	4	evm.model.LG11.1331	Protein SLOW GREEN 1, chloroplastic OS=Arabidopsis thaliana OX=3702 GN=SG1 PE=1 SV=1
ortholog14813	0.0027374	3	evm.model.LG03.1451	DNA topoisomerase 1 OS=Rickettsia felis (strain ATCC VR-1525 / URRWXCal2) OX=315456 GN=topA PE=3 SV=1
ortholog01906	0.0048762	3	evm.model.LG11.801	Shikimate O-hydroxycinnamoyltransferase OS=Nicotiana tabacum OX=4097 GN=HST PE=1 SV=1
ortholog01771	0.0397962	1	evm.model.LG03.1455	Pentatricopeptide repeat-containing protein At4g20090 OS=Arabidopsis thaliana OX=3702 GN=EMB1025 PE=3 SV=1
ortholog07856	0.0030996	1	evm.model.LG07.2270	Protein TPLATE OS=Arabidopsis thaliana OX=3702 GN=TPLATE PE=1 SV=1
ortholog01768	0.049791	1	evm.model.Contig1.40	Histone deacetylase 6 OS=Arabidopsis thaliana OX=3702 GN=HDA6 PE=1 SV=1
ortholog02986	0.0273032	1	evm.model.LG08.2012	PIN2/TERF1-interacting telomerase inhibitor 1 OS=Homo sapiens OX=9606 GN=PINX1 PE=1 SV=2
ortholog06205	0.0179918	5	evm.model.LG04.1690	Beta-galactosidase 8 OS=Arabidopsis thaliana OX=3702 GN=BGAL8 PE=2 SV=2
ortholog14768	0.0427751	1	evm.model.LG02.2032	Protein-ribulosamine 3-kinase, chloroplastic OS=Arabidopsis thaliana OX=3702 GN=At3g61080 PE=1 SV=2
ortholog06750	0.0351813	1	evm.model.LG01.1146	Histone-lysine N-methyltransferase ASHR3 OS=Arabidopsis thaliana OX=3702 GN=ASHR3 PE=1 SV=1
ortholog21377	0.0081235	3	evm.model.LG11.1960	ABC transporter G family member 5 OS=Arabidopsis thaliana OX=3702 GN=ABCG5 PE=2 SV=1
ortholog07183	0.0272783	2	evm.model.LG11.496	DEAD-box ATP-dependent RNA helicase 27 OS=Oryza sativa subsp. japonica OX=39947 GN=Os03g0802700 PE=3 SV=1
ortholog09026	0.0043311	1	evm.model.LG09.1036	Mitotic checkpoint serine/threonine-protein kinase BUB1 OS=Arabidopsis thaliana OX=3702 GN=BUB1 PE=1 SV=1
ortholog05497	0.0460302	2	evm.model.LG08.790	Protein IQ-DOMAIN 1 OS=Arabidopsis thaliana OX=3702 GN=IQD1 PE=1 SV=1
ortholog08710	0.0084743	3	evm.model.LG05.195	Lipoamide acyltransferase component of branched-chain alpha-keto acid dehydrogenase complex, mitochondrial OS=Arabidopsis thaliana OX=3702 GN=BCE2 PE=1 SV=1
ortholog01596	0.0130192	3	evm.model.LG01.846	Protein S-acyltransferase 24 OS=Arabidopsis thaliana OX=3702 GN=PAT24 PE=2 SV=1
ortholog04034	0.0113294	1	evm.model.LG02.2052	CRS2-associated factor 1, mitochondrial OS=Arabidopsis thaliana OX=3702 GN=At4g31010 PE=2 SV=1
ortholog14908	0.0002171	3	evm.model.LG08.482	6-hydroxynicotinate 3-monooxygenase OS=Pseudomonas fluorescens OX=294 GN=nicC PE=1 SV=1
ortholog16091	0.018697	1	evm.model.LG02.1207	DUF21 domain-containing protein At4g33700 OS=Arabidopsis thaliana OX=3702 GN=CBSDUF6 PE=1 SV=1
ortholog08162	0.0040577	2	evm.model.LG09.1499	Fatty acid hydroperoxide lyase, chloroplastic OS=Solanum lycopersicum OX=4081 GN=HPL PE=1 SV=1
ortholog01311	0.0010964	2	evm.model.LG07.262	NAD-dependent protein deacylase SRT2 OS=Arabidopsis thaliana OX=3702 GN=SRT2 PE=2 SV=1
ortholog01089	0.0050982	1	evm.model.LG04.971	Protein JASON OS=Arabidopsis thaliana OX=3702 GN=JASON PE=2 SV=1
ortholog13535	0.022935	1	evm.model.LG02.1504	Probable receptor-like serine/threonine-protein kinase At4g34500 OS=Arabidopsis thaliana OX=3702 GN=At4g34500 PE=2 SV=1
ortholog03567	0.0408132	1	evm.model.LG01.2270	ABC transporter A family member 1 OS=Arabidopsis thaliana OX=3702 GN=ABCA1 PE=2 SV=2
ortholog04344	0.0028076	4	evm.model.LG03.1383	Pentatricopeptide repeat-containing protein At4g01570 OS=Arabidopsis thaliana OX=3702 GN=At4g01570 PE=2 SV=1
ortholog19860	0.0342414	1	evm.model.LG11.784	
ortholog13244	0.0177403	3	evm.model.LG03.1950	Quinone oxidoreductase PIG3 OS=Homo sapiens OX=9606 GN=TP53I3 PE=1 SV=2
ortholog07767	0.0091873	4	evm.model.LG01.2631	Derlin-1 OS=Arabidopsis thaliana OX=3702 GN=DER1 PE=2 SV=1
ortholog16204	0.0005053	1	evm.model.LG01.528	E3 ubiquitin-protein ligase UPL7 OS=Arabidopsis thaliana OX=3702 GN=UPL7 PE=2 SV=1
ortholog01838	0.0047437	1	evm.model.LG01.720	Testis-expressed protein 10 homolog OS=Danio rerio OX=7955 GN=tex10 PE=2 SV=2
ortholog01274	0.0006672	3	evm.model.LG11.1260	Nitrate regulatory gene2 protein OS=Arabidopsis thaliana OX=3702 GN=NRG2 PE=1 SV=1
ortholog08317	0.0382537	2	evm.model.LG03.1808	Tyrosyl-DNA phosphodiesterase 1 OS=Arabidopsis thaliana OX=3702 GN=TDP1 PE=1 SV=1
ortholog05626	0.0344695	2	evm.model.LG10.422	Protein CHROMATIN REMODELING 19 OS=Arabidopsis thaliana OX=3702 GN=ETL1 PE=1 SV=1
ortholog11232	1.22E-05	7	evm.model.LG01.2212	DNA-(apurinic or apyrimidinic site) lyase, chloroplastic OS=Arabidopsis thaliana OX=3702 GN=ARP PE=1 SV=2
ortholog04489	0.0125144	2	evm.model.LG08.419	DNA cross-link repair 1A protein OS=Gallus gallus OX=9031 GN=DCLRE1A PE=1 SV=1
ortholog11064	0.0082551	5	evm.model.LG06.413	Phosphoinositide phosphatase SAC1 OS=Arabidopsis thaliana OX=3702 GN=SAC1 PE=1 SV=1
ortholog10353	0.002954	1	evm.model.LG10.351	Magnesium transporter MRS2-3 OS=Arabidopsis thaliana OX=3702 GN=MRS2-3 PE=2 SV=1
ortholog09460	0.033363	1	evm.model.LG07.43	DNA (cytosine-5)-methyltransferase 1 OS=Arabidopsis thaliana OX=3702 GN=DMT1 PE=1 SV=1
ortholog12264	0.0062221	2	evm.model.LG03.2595	Mannosyl-oligosaccharide 1,2-alpha-mannosidase MNS3 OS=Arabidopsis thaliana OX=3702 GN=MNS3 PE=1 SV=1
ortholog06527	0.0007685	2	evm.model.LG07.1482	Elongator complex protein 5 OS=Arabidopsis thaliana OX=3702 GN=ELP5 PE=1 SV=1
ortholog01870	5.43E-07	12	evm.model.LG11.1175	LysM domain receptor-like kinase 3 OS=Arabidopsis thaliana OX=3702 GN=LYK3 PE=2 SV=1
ortholog16087	0.0129833	1	evm.model.LG04.483	Neutral ceramidase 1 OS=Arabidopsis thaliana OX=3702 GN=NCER1 PE=2 SV=1
ortholog17966	0.0163047	2	evm.model.LG02.969	ATP-dependent DNA helicase Q-like 4A OS=Arabidopsis thaliana OX=3702 GN=RECQL4A PE=2 SV=1
ortholog01145	0.0080727	3	evm.model.LG02.2102	Pentatricopeptide repeat-containing protein At1g01970 OS=Arabidopsis thaliana OX=3702 GN=At1g01970 PE=2 SV=1
ortholog07157	0.0193547	1	evm.model.LG02.1200	Proline iminopeptidase OS=Arabidopsis thaliana OX=3702 GN=PIP PE=2 SV=3
ortholog14420	0.0032149	4	evm.model.LG11.1637	Aldose 1-epimerase OS=Pongo abelii OX=9601 GN=GALM PE=2 SV=1
ortholog05023	0.000867	2	evm.model.LG03.1752	tRNA wybutosine-synthesizing protein 2/3/4 OS=Arabidopsis thaliana OX=3702 GN=At4g04670 PE=2 SV=1
ortholog08154	0.0002583	8	evm.model.LG07.322	Probable inactive ATP-dependent zinc metalloprotease FTSHI 4, chloroplastic OS=Arabidopsis thaliana OX=3702 GN=FTSHI4 PE=1 SV=1
ortholog09213	0.0093065	1	evm.model.LG05.1588	3-isopropylmalate dehydratase large subunit, chloroplastic OS=Arabidopsis thaliana OX=3702 GN=IIL1 PE=1 SV=1
ortholog09109	0.0072525	3	evm.model.LG01.2580	Probable acyl-[acyl-carrier-protein]--UDP-N-acetylglucosamine O-acyltransferase, mitochondrial OS=Arabidopsis thaliana OX=3702 GN=LPXA PE=1 SV=1
ortholog03940	0.0082431	3	evm.model.LG03.579	Uroporphyrinogen decarboxylase, chloroplastic OS=Nicotiana tabacum OX=4097 GN=DCUP PE=1 SV=1
ortholog08042	0.0354568	2	evm.model.LG02.150	Exosome complex component RRP42 OS=Homo sapiens OX=9606 GN=EXOSC7 PE=1 SV=3
ortholog11550	0.0002	14	evm.model.LG04.962	Glutathionyl-hydroquinone reductase YqjG OS=Escherichia coli (strain K12) OX=83333 GN=yqjG PE=1 SV=1
ortholog02465	0.0189089	6	evm.model.LG07.2355	Nodulation receptor kinase OS=Pisum sativum OX=3888 GN=NORK PE=1 SV=1
ortholog08410	0.0119412	1	evm.model.LG09.1915	Cinnamoyl-CoA reductase-like SNL6 OS=Oryza sativa subsp. japonica OX=39947 GN=SNL6 PE=3 SV=1
ortholog14269	0.0001492	6	evm.model.LG06.106	Methionine aminotransferase OS=Escherichia coli (strain K12) OX=83333 GN=ybdL PE=1 SV=1
ortholog20277	0.0108613	5	evm.model.LG01.2690	Elongation factor Ts OS=Synechococcus sp. (strain RCC307) OX=316278 GN=tsf PE=3 SV=1
ortholog05971	0.0409475	1	evm.model.LG02.225	CSC1-like protein At3g54510 OS=Arabidopsis thaliana OX=3702 GN=At3g54510 PE=3 SV=1
ortholog13001	0.0003976	6	evm.model.LG11.554	Kinesin-like protein NACK1 OS=Nicotiana tabacum OX=4097 GN=NACK1 PE=1 SV=1
ortholog00692	0.0270552	2	evm.model.LG05.512	Hsp70-Hsp90 organizing protein 2 OS=Arabidopsis thaliana OX=3702 GN=HOP2 PE=1 SV=1
ortholog18729	0.0455463	6	evm.model.LG08.1693	Cytokinin dehydrogenase 7 OS=Arabidopsis thaliana OX=3702 GN=CKX7 PE=1 SV=1
ortholog08090	0.0244342	1	evm.model.LG11.462	Agmatine deiminase OS=Arabidopsis thaliana OX=3702 GN=AIH PE=1 SV=2
ortholog03430	0.0236906	2	evm.model.LG11.870	F-box/LRR-repeat protein 14 OS=Homo sapiens OX=9606 GN=FBXL14 PE=1 SV=1
ortholog00997	1.78E-07	9	evm.model.LG02.324	DNA gyrase subunit A, chloroplastic/mitochondrial OS=Nicotiana benthamiana OX=4100 GN=GYRA PE=2 SV=1
ortholog00149	0.0221075	3	evm.model.LG10.1366	Probable protein S-acyltransferase 22 OS=Arabidopsis thaliana OX=3702 GN=PAT22 PE=2 SV=2
ortholog05606	0.0057634	2	evm.model.LG10.1838	Photosynthetic NDH subunit of lumenal location 5, chloroplastic OS=Arabidopsis thaliana OX=3702 GN=PNSL5 PE=1 SV=1
ortholog15187	0.0001468	3	evm.model.LG07.439	Ubiquitin carboxyl-terminal hydrolase 26 OS=Oryza sativa subsp. indica OX=39946 GN=UBP26 PE=3 SV=1
ortholog00741	1.10E-05	6	evm.model.LG08.1826	Snurportin-1 OS=Gallus gallus OX=9031 GN=SNUPN PE=2 SV=1
ortholog06835	0.004941	1	evm.model.LG01.2605	Histone acetyltransferase type B catalytic subunit OS=Arabidopsis thaliana OX=3702 GN=HAG2 PE=2 SV=1
ortholog19448	0.0068965	1	evm.model.LG11.155	Lysine-specific demethylase REF6 OS=Arabidopsis thaliana OX=3702 GN=REF6 PE=1 SV=1
ortholog14123	0.0376921	1	evm.model.LG07.1377	GTPase ERA-like, chloroplastic OS=Arabidopsis thaliana OX=3702 GN=At5g66470 PE=2 SV=1
ortholog11953	0.002242	3	evm.model.LG04.439	Chaperone protein DnaJ OS=Trichodesmium erythraeum (strain IMS101) OX=203124 GN=dnaJ PE=3 SV=1
ortholog18862	0.0042474	1	evm.model.LG01.2765	DnaJ homolog subfamily C GRV2 OS=Arabidopsis thaliana OX=3702 GN=GRV2 PE=1 SV=1
ortholog08730	0.000804	2	evm.model.LG02.2147	Tubulin-folding cofactor D OS=Arabidopsis thaliana OX=3702 GN=TFCD PE=2 SV=1
ortholog13238	0.0009829	3	evm.model.LG03.2704	Fumarylacetoacetase OS=Arabidopsis thaliana OX=3702 GN=FAH PE=1 SV=1
ortholog06730	0.0291422	1	evm.model.LG07.1672	ABC transporter F family member 4 OS=Arabidopsis thaliana OX=3702 GN=ABCF4 PE=2 SV=1
ortholog02730	0.047447	1	evm.model.LG02.2266	Peptide chain release factor PrfB2, chloroplastic OS=Arabidopsis thaliana OX=3702 GN=PRFB2 PE=3 SV=1
ortholog06493	0.0054236	3	evm.model.LG11.1004	CCAAT/enhancer-binding protein zeta OS=Mus musculus OX=10090 GN=Cebpz PE=1 SV=2
ortholog13045	0.0018596	4	evm.model.LG07.2179	Polyribonucleotide nucleotidyltransferase 2, mitochondrial OS=Arabidopsis thaliana OX=3702 GN=PNP2 PE=2 SV=1
ortholog00793	1.99E-06	4	evm.model.LG07.657	Ribosome biogenesis protein TSR3 homolog OS=Mus musculus OX=10090 GN=Tsr3 PE=1 SV=1
ortholog04740	0.0039688	2	evm.model.LG02.2635	Integrator complex subunit 3 homolog OS=Dictyostelium discoideum OX=44689 GN=ints3 PE=3 SV=1
ortholog15652	0.0014299	1	evm.model.LG07.1505	RAB11-binding protein RELCH OS=Mus musculus OX=10090 GN=Relch PE=1 SV=1
ortholog04591	0.0130153	2	evm.model.LG03.2236	Plant-specific TFIIB-related protein PTF2 OS=Arabidopsis thaliana OX=3702 GN=PTF2 PE=1 SV=1
ortholog00512	0.0072238	1	evm.model.LG04.1426	Protein translocase subunit SECA1, chloroplastic OS=Arabidopsis thaliana OX=3702 GN=SECA1 PE=1 SV=2
ortholog08512	0.0033851	2	evm.model.LG04.1312	Poly [ADP-ribose] polymerase 2-A OS=Oryza sativa subsp. japonica OX=39947 GN=PARP2-A PE=3 SV=2
ortholog03976	1.60E-05	15	evm.model.LG10.145	Pentatricopeptide repeat-containing protein At5g18390, mitochondrial OS=Arabidopsis thaliana OX=3702 GN=At5g18390 PE=2 SV=2
ortholog11319	0.0362671	3	evm.model.LG01.1222	APO protein 2, chloroplastic OS=Arabidopsis thaliana OX=3702 GN=APO2 PE=2 SV=1
ortholog03956	0.0055243	3	evm.model.LG11.519	Pre-mRNA-splicing factor SPF27 homolog OS=Arabidopsis thaliana OX=3702 GN=MOS4 PE=1 SV=1

Supplementary Table S16. KEGG enrichment analysis of the DEGs in different clusters

KEGG pathway	Ko ID	Cluter frequency	*p* -value	Corrected *p* -value	Count
**Cluster 1**					
Photosynthesis	ko00195	2.95%	1.51E-22	1.99E-20	39
Biosynthesis of secondary metabolites	ko01110	34.09%	1.10E-11	1.45E-09	450
Photosynthesis - antenna proteins	ko00196	1.29%	1.14E-11	1.51E-09	17
Porphyrin and chlorophyll metabolism	ko00860	2.65%	1.90E-11	2.51E-09	35
Benzoxazinoid biosynthesis	ko00402	1.52%	6.90E-09	9.11E-07	20
Metabolic pathways	ko01100	50.38%	2.11E-07	2.79E-05	665
Glyoxylate and dicarboxylate metabolism	ko00630	2.65%	4.70E-07	6.20E-05	35
Carotenoid biosynthesis	ko00906	2.12%	6.64E-07	8.76E-05	28
Carbon fixation in photosynthetic organisms	ko00710	2.50%	4.33E-06	5.71E-04	33
Carbon metabolism	ko01200	5.98%	8.09E-05	1.07E-02	79
Tropane, piperidine and pyridine alkaloid biosynthesis	ko00960	1.52%	1.02E-04	1.35E-02	20
Riboflavin metabolism	ko00740	0.98%	6.12E-04	8.08E-02	13
**Cluster 2**					
Phenylpropanoid biosynthesis	ko00940	10.08%	1.79E-05	2.09E-03	50
Amino sugar and nucleotide sugar metabolism	ko00520	5.24%	4.03E-05	4.72E-03	26
**Cluster 3**					
Plant-pathogen interaction	ko04626	12.45%	2.54E-05	2.95E-03	61
Mismatch repair	ko03430	2.65%	1.90E-04	2.20E-02	13
RNA transport	ko03013	5.92%	2.58E-04	3.00E-02	29
**Cluster 4**					
Plant-pathogen interaction	ko04626	14.33%	1.31E-11	1.61E-09	103
ABC transporters	ko02010	3.34%	3.08E-04	3.79E-02	24
**Cluster 5**					
Protein processing in endoplasmic reticulum	ko04141	7.77%	3.28E-05	3.97E-03	37
**Cluster 6**					
Biosynthesis of secondary metabolites	ko01110	35.49%	6.62E-10	8.28E-08	192
Metabolic pathways	ko01100	52.50%	2.25E-05	2.81E-03	284
Monoterpenoid biosynthesis	ko00902	2.40%	8.71E-05	1.09E-02	13
Cutin, suberine and wax biosynthesis	ko00073	2.40%	2.53E-04	3.16E-02	13
**Cluster 7**					
Peroxisome	ko04146	5.47%	1.96E-05	2.19E-03	17
Riboflavin metabolism	ko00740	2.25%	1.29E-04	1.45E-02	7
Circadian rhythm - plant	ko04712	3.86%	3.71E-04	4.15E-02	12
**Cluster 8**					
MAPK signaling pathway - plant	ko04016	6.92%	2.97E-04	3.44E-02	34
**Cluster 9**					
MAPK signaling pathway - plant	ko04016	6.78%	3.49E-06	4.40E-04	58
Phenylpropanoid biosynthesis	ko00940	9.11%	3.61E-06	4.55E-04	78
Other types of O-glycan biosynthesis	ko00514	2.22%	8.89E-06	1.12E-03	19

Supplementary Table S17. The expression of corresponding candidate genes related to anthraquinone and iridoid biosynthesis pathways

Gene ID	Description	Function	Leaf	Stalk	AR	TR	SR
**Shikimate pathway**							
evm.model.LG02.1109	DAHPS	3-Deoxy-7-phosphoheptulonate synthase	111.09	69.55	45.8033	65.28	89.63
evm.model.LG06.1541	DAHPS	3-Deoxy-7-phosphoheptulonate synthase	632.057	831.453	517.44	381.35	126.607
evm.model.LG07.845	DAHPS	3-Deoxy-7-phosphoheptulonate synthase	173.207	79.9033	67.1933	142.197	146.783
evm.model.Contig51.3	DHQS	3-Dehydroquinate synthase	10.5033	6.58333	3.3	1.19	2.12333
evm.model.LG07.1258	DHQS	3-Dehydroquinate synthase	118.827	92.13	72.45	88.74	90.9567
evm.model.LG07.1530	DHQS	3-Dehydroquinate synthase	0.02333	0.10333	0.03	1.7	1.36667
evm.model.LG02.2521	DHQ/SDH	3-Dehydroquinate dehydratase/shikimate dehydrogenase	3.71667	21.9733	6.24	5.41667	0.42667
evm.model.LG04.1237	DHQ/SDH	3-Dehydroquinate dehydratase/shikimate dehydrogenase	23.72	25.1933	3.41667	1.33333	18.4433
evm.model.LG05.301	DHQ/SDH	3-Dehydroquinate dehydratase/shikimate dehydrogenase	48.75	64.7533	41.6433	24.54	42.0033
evm.model.LG08.1157	DHQ/SDH	3-Dehydroquinate dehydratase/shikimate dehydrogenase	0	0.06333	0	0	0
evm.model.LG06.238	SK	Shikimate kinase	17.2333	8.25667	3.53667	4.44333	5.34667
evm.model.LG07.2334	SK	Shikimate kinase	69.2633	17.1333	11.1733	18.09	17.6767
evm.model.LG07.563	SK	Shikimate kinase	126.99	51.41	31.06	43.1867	52.6633
evm.model.LG08.1263	SK	Shikimate kinase	37.26	9.92333	3.86667	6.53	8.7
evm.model.LG02.2136	EPSPS	3-Phosphoshikimate 1-carboxyvinyltransferase	42.9133	73.46	56.9567	39.55	38.19
evm.model.LG03.1168	CS	Chorismate synthase	207.643	240.74	180.717	146.86	103.173
evm.model.LG10.1245	PHYLLO	2-Succinyl-5-enolpyruvyl-6-hydroxy-3-cyclohexene-1-carboxylate synthase	4.16667	23.66	27.4333	39.8833	106.607
evm.model.LG11.1662	ICS	Isochorismate synthase	4.79667	7.91667	6.11	3.49333	29.6133
evm.model.LG03.1376	MenE	O-succinylbenzoyl-CoA ligase	15.6133	5.22333	7.44333	7.7	4.39
evm.model.LG10.1634	MenB	1,4-Dihydroxy-2-naphthoyl-CoA synthase	71.63	29.9067	0.10333	0.03667	0.04
evm.model.LG10.1246	MenB	1,4-Dihydroxy-2-naphthoyl-CoA synthase	53.8533	156.363	418.63	628.773	465.393
evm.model.LG08.1736	MenI	1,4-Dihydroxy-2-naphthoyl-CoA hydrolase	92.12	66.0433	40.1967	39.3533	53.08
evm.model.LG11.2035	MenI	1,4-Dihydroxy-2-naphthoyl-CoA hydrolase	0	0.23333	1.64333	5.15333	3.67
evm.model.Contig1.15	MenI	1,4-Dihydroxy-2-naphthoyl-CoA hydrolase	0.30667	0.67333	2.49667	1.84	2.96
evm.model.LG04.1501	MenI	1,4-Dihydroxy-2-naphthoyl-CoA hydrolase	41.4767	73.0433	93.6433	48.6967	136.163
**MEP pathway**							
evm.model.LG01.1061	DXS	1-Deoxy-D-xylulose-5-phosphate synthase	0.04	33.6467	55.2567	11.81	4.28
evm.model.LG05.311	DXS	1-Deoxy-D-xylulose-5-phosphate synthase	32.2467	82.3333	9.89	12.4267	15.7133
evm.model.LG07.962	DXS	1-Deoxy-D-xylulose-5-phosphate synthase	86.96	55.2367	107.097	67.54	42.3033
evm.model.LG08.474	DXS	1-Deoxy-D-xylulose-5-phosphate synthase	14.7033	11.4033	6.15667	7.09	7.81
evm.model.LG11.1881	DXR	1-Deoxy-D-xylulose-5-phosphate reductoisomerase	124.733	180.203	122.873	137.403	86.18
evm.model.LG02.888	CMS	2-C-Methyl-D-erythritol 4-phosphate cytidylyltransferase	57.9333	39.2167	24.01	20.1133	20.0267
evm.model.LG04.2152	CMK	4-Diphosphocytidyl-2-C-methyl-D-erythritol kinase	36.3333	49.92	40.5533	32.2133	47.42
evm.model.LG08.2015	MCS	2-C-Methyl-D-erythritol 2,4-cyclodiphosphate Synthase	534.613	386.58	317.69	241.05	221.633
evm.model.LG04.1699	HDS	(E)-4-Hydroxy-3-methylbut-2-enyl-diphosphate synthase	370.023	177.153	182.537	103.427	169.1
evm.model.LG02.1467	HDR	4-Hydroxy-3-methylbut-2-enyl diphosphate reductase	1042.81	528.423	370.8	254.487	220.49
**MVA pathway**							
evm.model.LG03.2636	ACAT	Acetyl-CoA C-acetyltransferase	36.7067	34.9667	16.98	17.6733	37.8667
evm.model.LG09.1801	ACAT	Acetyl-CoA C-acetyltransferase	128.053	273.493	341.857	227.277	78.6533
evm.model.LG08.949	HMGS	Hydroxymethylglutaryl-CoA synthase	121.913	140.627	135.483	70.97	35.45
evm.model.LG02.996	HMGR	Hydroxymethylglutaryl-CoA reductase	7.26	13.2633	6.19	5.74333	9.46333
evm.model.LG06.1311	HMGR	Hydroxymethylglutaryl-CoA reductase	5.87667	18.26	13.3167	36.4867	41.9167
evm.model.LG07.1127	HMGR	Hydroxymethylglutaryl-CoA reductase	97.01	221.877	162.94	95.6667	133.907
evm.model.LG02.2445	MVK	Mevalonate kinase	19.7	35.4933	30.8733	21.2733	17.06
evm.model.Contig38.13	PMK	Phosphomevalonate kinase	0.05333	0.2	0.10667	0.02333	0
evm.model.LG08.1230	PMK	Phosphomevalonate kinase	4.80333	10.18	8.51	25.86	13.1067
evm.model.Contig38.11	PMK	Phosphomevalonate kinase	16.8667	27.68	23.71	8.08333	9.98667
evm.model.LG03.996	MVD	Methyl parathion hydrolase	50.1633	106.02	85.4933	51.9967	61.1067
evm.model.LG03.1323	IDI	Isopentenyl-diphosphate delta-isomerase	126.317	307.68	1017.07	413.83	206.497
evm.model.LG09.1874	IDI	Isopentenyl-diphosphate delta-isomerase	0	0	0	0	0
**Iridoid biosynthesis pathway**							
evm.model.LG10.419	GPPS	Geranyl-diphosphate synthase	23.2833	19.34	15.7833	15.8567	20.44
evm.model.LG05.1964	GPPS	Geranyl-diphosphate synthase	7.98667	10.9467	8.52667	10.1767	28.85
evm.model.LG09.1256	GES	Geraniol synthase	3.21333	98.0367	76.69	13.8633	3.99667
evm.model.LG10.368	G10H	Geraniol 10-hydroxylase	0.24667	0.04	0	0	0
evm.model.Contig20.14	G10H	Geraniol 10-hydroxylase	0.34333	0.06333	0	0	0
evm.model.LG07.1780	G10H	Geraniol 10-hydroxylase	1.20333	3.36667	21.9067	21.5167	46.21
evm.model.LG07.1781	G10H	Geraniol 10-hydroxylase	59.3733	208.39	191.573	71.6767	17.6233
evm.model.LG01.1450	G10H	Geraniol 10-hydroxylase	0	0	0	0	0
evm.model.LG02.2073	G10H	Geraniol 10-hydroxylase	0	0	0	0	0
evm.model.LG02.2075	G10H	Geraniol 10-hydroxylase	0	0.09	4.18667	0.28333	9.85667
evm.model.LG02.2077	G10H	Geraniol 10-hydroxylase	0.33333	1.03	10.4233	18.5733	5.6
evm.model.LG02.2078	G10H	Geraniol 10-hydroxylase	14.35	9.32333	2.49667	0.83	14.7567
evm.model.LG02.2079	G10H	Geraniol 10-hydroxylase	6.13667	7.92667	11.45	2.78667	3.98
evm.model.LG02.2080	G10H	Geraniol 10-hydroxylase	2.13667	2.23	2.38667	1.20667	2.09
evm.model.LG03.659	G10H	Geraniol 10-hydroxylase	0.39667	0.73333	1.00667	0.17333	0.02333
evm.model.LG08.1504	G10H	Geraniol 10-hydroxylase	0	0	0	0	0
evm.model.LG08.874	G10H	Geraniol 10-hydroxylase	42.1	4.71667	1.02333	0.61667	2.71333
evm.model.LG10.709	G10H	Geraniol 10-hydroxylase	0	0	0	0	0
evm.model.LG07.969	10HGO	10-Hydroxygeraniol dehydrogenase	0	0	0	0	0
evm.model.LG07.973	10HGO	10-Hydroxygeraniol dehydrogenase	0	0	0	0	0
evm.model.LG07.974	10HGO	10-Hydroxygeraniol dehydrogenase	8.60333	10.77	9.53	7.30667	2.53667
evm.model.LG07.976	10HGO	10-Hydroxygeraniol dehydrogenase	2.47	6.26667	4.64667	2.44667	2.28333
evm.model.LG07.977	10HGO	10-Hydroxygeraniol dehydrogenase	2.85	35.8967	10.1267	5.12667	17.44
evm.model.LG07.982	10HGO	10-Hydroxygeraniol dehydrogenase	799.987	692.037	214.893	341.263	271.977
evm.model.LG06.683	IS	Iridoid synthase	0.26333	106.3	37.9667	9.91333	7.85667
evm.model.LG06.684	IS	Iridoid synthase	0.09667	50.15	39.5	7.36333	3.07667
evm.model.LG04.537	IS	Iridoid synthase	0.01667	0	0.05	0	0
evm.model.LG01.550	7-DLS/CYP76A26	7-Deoxyloganetic acid synthase	0	0	0.29333	0.82	0.58
evm.model.LG01.551	7-DLS/CYP76A26	7-Deoxyloganetic acid synthase	0.05333	2.66333	5.97	1.72667	0.52333
evm.model.LG01.552	7-DLS/CYP76A26	7-Deoxyloganetic acid synthase	1.58	166.607	123.733	27.7033	15.1267
evm.model.LG03.1320	7-DLS/CYP76A26	7-Deoxyloganetic acid synthase	0	0.09667	0.01333	0.02667	0
evm.model.LG03.1054	7-DLGT/UGT8	7-Deoxyloganetic acid glucosyltransferase	0.08333	0.19667	0.17	0.05	0.16667
evm.model.LG03.715	7-DLGT/UGT8	7-Deoxyloganetic acid glucosyltransferase	19.9367	75.1667	53.6467	14.7533	11.1267
evm.model.LG03.716	7-DLGT/UGT8	7-Deoxyloganetic acid glucosyltransferase	33.15	3.61333	1.06667	6.05333	3.48
evm.model.LG03.717	7-DLGT/UGT8	7-Deoxyloganetic acid glucosyltransferase	0.03	1.10667	2.33667	0.17667	0.36667
evm.model.LG03.718	7-DLGT/UGT8	7-Deoxyloganetic acid glucosyltransferase	6.93333	3.39333	9.52667	9.15333	24.1167
evm.model.LG03.719	7-DLGT/UGT8	7-Deoxyloganetic acid glucosyltransferase	4.02	4.44	20.41	4.57667	4.49667
evm.model.LG03.720	7-DLGT/UGT8	7-Deoxyloganetic acid glucosyltransferase	16.7167	14.7033	13.3133	5.64	5.21
evm.model.LG03.721	7-DLGT/UGT8	7-Deoxyloganetic acid glucosyltransferase	0.68667	0.47667	0.29667	0.17	0.1
evm.model.LG03.723	7-DLGT/UGT8	7-Deoxyloganetic acid glucosyltransferase	2.52	17.9267	13.97	6.28667	9.99
evm.model.LG03.724	7-DLGT/UGT8	7-Deoxyloganetic acid glucosyltransferase	0.28667	0.16	0	0	0
evm.model.LG03.725	7-DLGT/UGT8	7-Deoxyloganetic acid glucosyltransferase	36.9633	31.84	3.69	4.36333	9.57
evm.model.LG07.357	7-DLGT/UGT8	7-Deoxyloganetic acid glucosyltransferase	31.9533	8.82	5.08	11.9367	33.9

Supplementary Table S18. Identification of TPS gene families in *M. officinalis*

Symbol	Gene ID	Group	Length	E-value	Leaf	Stalk	AR	TR	SR	KO ID	KEGG	SwissProt
MoTPS01	evm.model.LG02.46	a	487	6.3E-86	0.02	1.356667	18.94	0.41	2.056667	K15804	5-Epiaristolochene synthase	Viridiflorene synthase
MoTPS02	evm.model.LG02.47	a	226	2.3E-14	0.396667	0.416667	0.26	0.066667	0.013333	K14182	Vetispiradiene synthase	Viridiflorene synthase
MoTPS03	evm.model.LG02.48	a	221	3.6E-37	0.58	0.58	0.703333	0.346667	0.08	K14182	Vetispiradiene synthase	Vetispiradiene synthase
MoTPS04	evm.model.LG02.49	a	117	5.8E-15	0	0	0	0	0	K14182	Vetispiradiene synthase	Viridiflorene synthase
MoTPS05	evm.model.LG02.50	a	206	1.6E-42	0.046667	0.03	0.083333	0	0	K15804	5-Epiaristolochene synthase	5-Epi-aristolochene synthase
MoTPS06	evm.model.LG02.51	a	135	9.9E-16	0	0	0	0.043333	0	K14182	Vetispiradiene synthase	Viridiflorene synthase
MoTPS07	evm.model.LG02.53	a	348	5.3E-48	0.216667	0.18	0.413333	0.143333	0.016667	K15803	(-)-Germacrene D synthase	Germacrene A synthase
MoTPS08	evm.model.LG02.54	a	360	6E-48	0.496667	0.523333	0.736667	0.29	0.226667	K15803	(-)-Germacrene D synthase	Vetispiradiene synthase
MoTPS09	evm.model.LG02.55	a	314	5.9E-77	0.083333	0.02	0.09	0.04	0	K14182	Vetispiradiene synthase	Vetispiradiene synthase
MoTPS10	evm.model.LG02.57	a	556	9.1E-89	0.08	0.123333	3.403333	1.496667	0.78	K14182	Vetispiradiene synthase	Viridiflorene synthase
MoTPS11	evm.model.LG06.2129	a	701	2.1E-87	0.67	0.583333	1.2	0.473333	0.13	K14182	Vetispiradiene synthase	Vetispiradiene synthase
MoTPS12	evm.model.LG06.72	a	551	1.9E-88	0	0.043333	0.08	0	0	K14182	Vetispiradiene synthase	Viridiflorene synthase
MoTPS13	evm.model.LG08.377	a	614	8.9E-93	0.11	0.443333	0.303333	0.186667	0.07	K18117	Vetispiradiene synthase	Viridiflorene synthase
MoTPS14	evm.model.LG08.404	a	553	1.9E-92	0.09	0.39	0.293333	0.223333	0	K18117	Vetispiradiene synthase	Viridiflorene synthase
MoTPS15	evm.model.LG02.1143	b	567	2E-56	1.746667	1.063333	18.51	10.12	0.883333	K07385	1,8-Cineole synthase	Myrcene synthase
MoTPS16	evm.model.LG02.1145	b	392	1.4E-55	0.113333	0.04	0.006667	0.04	0	K07385	1,8-Cineole synthase	Terpene synthase 10
MoTPS17	evm.model.LG02.1146	b	530	1.6E-54	4.863333	7.45	2.646667	0.016667	11.80333	K07385	1,8-Cineole synthase	(+)-Alpha-pinene synthase
MoTPS18	evm.model.LG02.1147	b	217	2.3E-49	0	0.026667	0	0	0.03	K07385	1,8-Cineole synthase	Myrcene synthase
MoTPS19	evm.model.LG02.780	b	551	6.8E-84	0.06	0.31	0.026667	0	0	K07385	1,8-Cineole synthase	(-)-Alpha-terpineol synthase
MoTPS20	evm.model.LG02.781	b	610	1.5E-107	0.006667	0.71	5.473333	6.063333	2.19	K18108	(-)-Alpha-terpineol synthase	(-)-Alpha-terpineol synthase
MoTPS21	evm.model.LG03.1841	b	551	9.1E-91	9.053333	3.196667	0	0	0	K14173	Alpha-farnesene synthase	Alpha-farnesene synthase
MoTPS22	evm.model.LG03.1842	b	551	6.3E-93	34.81	109.18	0.59	0	0.196667	K14173	Alpha-farnesene synthase	Alpha-farnesene synthase
MoTPS23	evm.model.LG06.2062	b	535	6.9E-84	1.623333	1.13	0.823333	1.09	0.21	K07385	1,8-Cineole synthase	Terpene synthase 10
MoTPS24	evm.model.LG06.2064	b	566	3.1E-97	2.456667	4.256667	0	0	0.013333	K07385	1,8-Cineole synthase	(-)-Alpha-terpineol synthase
MoTPS25	evm.model.LG06.2066	b	313	4.1E-70	0.03	0.09	0	0.04	0.02	K07385	1,8-Cineole synthase	Terpene synthase 10
MoTPS26	evm.model.LG06.2067	b	184	3.6E-40	0	0.016667	0	0	0	K07385	1,8-Cineole synthase	Myrcene synthase
MoTPS27	evm.model.LG06.2070	b	576	5.8E-95	0.093333	0.63	0.01	0.006667	0.036667	K07385	1,8-Cineole synthase	(-)-Alpha-terpineol synthase
MoTPS28	evm.model.LG06.2071	b	597	8.2E-96	0.05	1.786667	0.01	0.12	0.496667	K07385	1,8-Cineole synthase	Myrcene synthase
MoTPS29	evm.model.LG01.1981	c	152	2.5E-16	0	0	0	0	0	K04120	Ent-copalyl diphosphate synthase	Copal-8-ol diphosphate hydratase
MoTPS30	evm.model.LG01.1985	c	731	4E-50	0	0.18	0.013333	0.023333	0	K04120	Ent-copalyl diphosphate synthase	Ent-copalyl diphosphate synthase
MoTPS31	evm.model.LG01.1988	c	808	4.9E-50	0	0.006667	0.11	0.223333	0	K04120	Ent-copalyl diphosphate synthase	Copal-8-ol diphosphate hydratase
MoTPS32	evm.model.LG04.1147	c	826	3.8E-52	0.07	0.626667	0.033333	0.023333	0.116667	K04120	Ent-copalyl diphosphate synthase	Ent-copalyl diphosphate synthase
MoTPS33	evm.model.LG09.16	e	824	5.3E-74	14.20333	9.06	3.403333	6.193333	18.86667	K04121	Ent-kaurene synthase	Ent-kaur-16-ene synthase
MoTPS34	evm.model.LG09.1847	e	542	4.1E-39	23.74333	11.24333	4.506667	0.01	0.01	K04121	Ent-kaurene synthase	Ent-kaur-16-ene synthase
MoTPS35	evm.model.LG03.1976	f	827	1.4E-61	16.72667	111.2433	119.4767	16.91667	3.21	K17982	Geranyllinalool synthase	S-Linalool synthase
MoTPS36	evm.model.LG03.1977	f	858	1E-60	0.396667	0.036667	0.013333	0.003333	0	K17982	Geranyllinalool synthase	S-Linalool synthase
MoTPS37	evm.model.LG03.1979	f	858	3.6E-61	0.213333	0.13	0.12	0.01	0.003333	K17982	Geranyllinalool synthase	S-Linalool synthase
MoTPS38	evm.model.LG03.1983	f	858	3.8E-61	0.056667	0.033333	0.013333	0	0	K17982	Geranyllinalool synthase	S-Linalool synthase
MoTPS39	evm.model.LG03.1847	g	579	5.7E-67	1.37	22.55667	0.41	0.043333	0	K14175	(3S,6E)-Nerolidol synthase	(3S,6E)-Nerolidol synthase 1
MoTPS40	evm.model.LG09.1256	g	587	5.5E-95	3.213333	98.03667	76.69	13.86333	3.996667	K20979	Geraniol synthase	Geraniol synthase
MoTPS41	evm.model.LG01.1980	-	778	4.4E-57	0.016667	2.366667	0.03	0	0	K04121	Ent-kaurene synthase	Cis-abienol synthase

Supplementary Table S19. The expression of corresponding candidate genes for polysaccharide biosynthesis in *M. officinalis*

Gene ID	Description	KO ID	Function	Leaf	Stalk	AR	TR	SR
evm.model.LG01.650	sacA	K01193	Beta-fructofuranosidase	16.5233	0.43667	0	0	0
evm.model.LG01.651	sacA	K01193	Beta-fructofuranosidase	0	0.00333	0	0	0
evm.model.LG01.652	sacA	K01193	Beta-fructofuranosidase	0	0.01333	0	0	0
evm.model.LG01.653	sacA	K01193	Beta-fructofuranosidase	0.07667	0.99	0.93	0.36333	0.32333
evm.model.LG03.433	sacA	K01193	Beta-fructofuranosidase	65.3167	361.78	322.96	407.55	372.833
evm.model.LG03.435	sacA	K01193	Beta-fructofuranosidase	5.29667	142.247	22.87	9.19	100.593
evm.model.LG08.156	sacA	K01193	Beta-fructofuranosidase	0.02667	0.34	0	1.35667	1.39
evm.model.LG08.847	sacA	K01193	Beta-fructofuranosidase	0.46	2.43	0.18667	0	0
evm.model.LG11.1097	sacA	K01193	Beta-fructofuranosidase	163.073	37.2967	6.88333	2.91	0.2
evm.model.LG03.1628	malZ	K01187	Alpha-glucosidase	70.59	7.3	5.00667	2.52	3.28667
evm.model.LG03.1631	malZ	K01187	Alpha-glucosidase	18.7867	18.9533	13.54	5.39667	1.82
evm.model.LG03.1642	malZ	K01187	Alpha-glucosidase	0.72	0.69333	0.23667	0.13	0.12667
evm.model.LG08.260	malZ	K01187	Alpha-glucosidase	0.14	0.13333	0.18667	0.78333	0.33667
evm.model.LG02.2055	scrK	K00847	Fructokinase	0.98667	1.11	0.14	0.1	0.01667
evm.model.LG03.372	scrK	K00847	Fructokinase	16.1067	46.1167	142.023	122.937	42.3567
evm.model.LG05.2194	scrK	K00847	Fructokinase	112.48	238.563	280.937	570.093	103.767
evm.model.LG06.1341	scrK	K00847	Fructokinase	1.05	0.71	2.50667	8.89	2.04333
evm.model.LG07.2035	scrK	K00847	Fructokinase	28.04	15.5767	2.80667	3.83667	19.6333
evm.model.LG08.718	scrK	K00847	Fructokinase	0.05667	0.23667	0.08	0	0
evm.model.LG07.1481	MPI	K01809	Mannose-6-phosphate isomerase	31.8833	77.8333	80.6833	90.33	37.92
evm.model.LG02.11	PMM	K17497	Phosphomannomutase	1.27333	1.82333	1.49333	10.9733	0
evm.model.LG02.17	PMM	K17497	Phosphomannomutase	1.03333	2.23333	1.86333	9.98333	0.07667
evm.model.LG02.1985	PMM	K17497	Phosphomannomutase	48.5033	42.3767	30.9933	41.9467	18.82
evm.model.LG02.21	PMM	K17497	Phosphomannomutase	5.36	5.77333	3.67	8.82333	0
evm.model.LG02.5	PMM	K17497	Phosphomannomutase	9.41333	9.22	6.24667	21.4733	0
evm.model.LG02.9	PMM	K17497	Phosphomannomutase	7.48	5.89667	6.12333	18.0033	0.11
evm.model.LG04.2317	PMM	K17497	Phosphomannomutase	0	0	0	0	0
evm.model.LG09.1655	PMM	K17497	Phosphomannomutase	2.96333	2.95333	2.92	0	0
evm.model.LG03.750	GMPP	K00966	Mannose-1-phosphate guanylyltransferase	61.9333	58.6833	94.0567	93.0933	51.7767
evm.model.LG07.1309	GMDS	K01711	GDP-mannose 4,6-dehydratase	23.1367	45.27	65.63	52.1	28.6567
evm.model.LG01.1118	TSTA3	K02377	GDP-L-fucose synthase	26.4767	33.9533	25.5367	27.5833	19.03
evm.model.LG01.2645	HK	K00844	Hexokinase	48.33	79.6667	192.29	163.93	80.5967
evm.model.LG01.2648	HK	K00844	Hexokinase	1.48667	2.36	5.53667	3.34667	5.63667
evm.model.LG02.68	HK	K00844	Hexokinase	0.04667	0.41333	0.05	0	0
evm.model.LG03.1985	HK	K00844	Hexokinase	0	0.12333	0	0	0
evm.model.LG03.2800	HK	K00844	Hexokinase	0	0	0	0	0
evm.model.LG04.2314	HK	K00844	Hexokinase	12.0467	13.2	11.28	17.96	53.97
evm.model.LG05.263	HK	K00844	Hexokinase	0	0	0	0	0
evm.model.LG06.1171	HK	K00844	Hexokinase	5.56	21.6267	9.06333	7.35333	0.77
evm.model.LG07.1572	HK	K00844	Hexokinase	0.07667	0.38667	0	0.02	0
evm.model.LG09.1928	HK	K00844	Hexokinase	21.3567	20.3633	18.2967	18.3533	19.46
evm.model.LG10.102	HK	K00844	Hexokinase	1.52	0.36	0	1.81333	14.7133
evm.model.LG11.1173	HK	K00844	Hexokinase	42.88	34.8467	18.8233	20.9733	23.1233
evm.model.Contig13.2	pgm	K01835	Phosphoglucomutase	100.177	108.62	79.0233	90.24	76.5433
evm.model.LG03.373	pgm	K01835	Phosphoglucomutase	69.9	37.5167	21.7267	23.65	35.43
evm.model.LG10.815	pgm	K01835	Phosphoglucomutase	19.8033	16.69	13.0033	15.0733	12.8367
evm.model.LG06.1038	GPI	K01810	Glucose-6-phosphate isomerase	69.5933	97.7233	176.053	352.567	38.72
evm.model.LG06.1468	GPI	K01810	Glucose-6-phosphate isomerase	132.71	134.007	101.62	95.17	124.407
evm.model.LG02.506	UGP2	K00963	UTP-glucose-1-phosphate uridylyltransferase	18.09	24.7667	13.11	15.77	25.17
evm.model.LG07.1060	UGP2	K00963	UTP-glucose-1-phosphate uridylyltransferase	138.073	133.107	127.22	114.297	79.7267
evm.model.LG06.1985	USP	K12447	UDP-sugar pyrophosphorylase	39.0233	34.3567	32.47	32.3067	39.1967
evm.model.LG07.1944	UGDH	K00012	UDP-glucose 6-dehydrogenase	156.793	348.167	614.247	376.23	251.697
evm.model.LG09.1402	UGDH	K00012	UDP-glucose 6-dehydrogenase	0.02667	0.08	0.00667	0	0
evm.model.LG09.1404	UGDH	K00012	UDP-glucose 6-dehydrogenase	0	0	0	0	0
evm.model.LG10.1337	UGDH	K00012	UDP-glucose 6-dehydrogenase	1.12667	6.45333	0.52667	0.07667	0
evm.model.LG11.1458	UGDH	K00012	UDP-glucose 6-dehydrogenase	15.22	26.78	15.09	4.99667	6.13333
evm.model.LG01.345	UXS1	K08678	UDP-glucuronate decarboxylase	29.13	57.7233	47.49	52.6933	44.35
evm.model.LG03.1685	UXS1	K08678	UDP-glucuronate decarboxylase	0.92333	4.25667	3.08333	5.58	1.42
evm.model.LG03.815	UXS1	K08678	UDP-glucuronate decarboxylase	130.233	157.167	214.59	124.847	121.08
evm.model.LG04.1549	UXS1	K08678	UDP-glucuronate decarboxylase	105.35	155.61	142.41	63.3833	65.2233
evm.model.LG05.205	UXS1	K08678	UDP-glucuronate decarboxylase	0.10667	7.32	2.22333	0.14333	0.03
evm.model.LG04.1834	AXS	K12449	UDP-apiose/xylose synthase	338.73	361.863	588.043	508.403	507.6
evm.model.LG03.1620	UXE	K12448	UDP-arabinose 4-epimerase	150.56	313.013	402.237	330.677	117.887
evm.model.LG09.2080	UXE	K12448	UDP-arabinose 4-epimerase	12.12	28.3667	8.69333	13.8	47.5933
evm.model.LG08.204	RHM	K12450	UDP-glucose 4,6-dehydratase	0	0	0	0	0
evm.model.LG09.648	RHM	K12450	UDP-glucose 4,6-dehydratase	313.86	575.527	821.703	349.52	585.147
evm.model.LG09.797	RHM	K12450	UDP-glucose 4,6-dehydratase	0.82	0.36	0.16333	0.20333	0
evm.model.LG08.944	UER1	K12451	3,5-Epimerase/4-reductase	183.43	116.367	107.347	68.15	51.71
evm.model.LG08.1915	GALE	K01784	UDP-glucose 4-epimerase	58.24	163.98	83.23	60.1533	156.583
evm.model.LG08.987	GALE	K01784	UDP-glucose 4-epimerase	54.4133	110.547	76.4467	78.5867	59.8167
evm.model.Contig15.9	GAE	K08679	UDP-glucuronate 4-epimerase	10.2533	30.2433	50.19	35.9967	3.36333
evm.model.LG01.1111	GAE	K08679	UDP-glucuronate 4-epimerase	364.717	523.997	249.293	183.06	759.907
evm.model.LG02.2134	GAE	K08679	UDP-glucuronate 4-epimerase	9.30333	29.1567	26.0367	17.0467	6.09
evm.model.LG05.1300	GAE	K08679	UDP-glucuronate 4-epimerase	5.02333	17.6933	33.5567	14.0933	0.28667
evm.model.LG08.877	GAE	K08679	UDP-glucuronate 4-epimerase	34.4867	57.0467	13.3967	15.49	28.1567

Supplementary Table S20. Expanded gene families related to active ingredient biosyathsis and sugar metabolism

Gene ID	Description	KO ID	Function	Leaf	Stalk	AR	TR	SR
**map00400 Phenylalanine, tyrosine and tryptophan biosynthesis**								
evm.model.Contig51.3	DHQS	K01735	3-Dehydroquinate synthase	10.5033	6.58333	3.3	1.19	2.12333
evm.model.LG07.1530	DHQS	K01735	3-Dehydroquinate synthase	0.02333	0.10333	0.03	1.7	1.36667
**map00900 Terpenoid backbone biosynthesis**								
evm.model.LG06.1001	GGPPS	K13789	Geranylgeranyl diphosphate synthase	1.02	6.62333	5.77	5.25667	8.84667
evm.model.LG06.1002	GGPPS	K13789	Geranylgeranyl diphosphate synthase	0	0	0.28667	0.01667	0.02667
evm.model.LG06.1003	GGPPS	K13789	Geranylgeranyl diphosphate synthase	0	0	0	0	0
evm.model.LG06.1004	GGPPS	K13789	Geranylgeranyl diphosphate synthase	25.73	19.7233	15.3767	13.64	6.27667
evm.model.LG07.1453	GGPPS	K13789	Geranylgeranyl diphosphate synthase	129.5	61.2	29.4467	12.78	9.07
evm.model.Contig14.62	chlP	K10960	Geranylgeranyl diphosphate	37.17	9.96667	0.21333	0.35667	0.04333
evm.model.LG02.158	chlP	K10960	Geranylgeranyl diphosphate	0.8	2.70333	1.54	2.77	2.48667
evm.model.LG11.1576	chlP	K10960	Geranylgeranyl diphosphate	36.23	8.03	0.22333	0.2	0
**map00902 Monoterpenoid biosynthesis**								
evm.model.LG02.1143	TPS-Cin	K07385	1,8-Cineole synthase	1.74667	1.06333	18.51	10.12	0.88333
evm.model.LG02.1145	TPS-Cin	K07385	1,8-Cineole synthase	0.11333	0.04	0.00667	0.04	0
evm.model.LG02.1146	TPS-Cin	K07385	1,8-Cineole synthase	4.86333	7.45	2.64667	0.01667	11.8033
evm.model.LG06.2062	TPS-Cin	K07385	1,8-Cineole synthase	1.62333	1.13	0.82333	1.09	0.21
evm.model.LG06.2064	TPS-Cin	K07385	1,8-Cineole synthase	2.45667	4.25667	0	0	0.01333
evm.model.LG06.2066	TPS-Cin	K07385	1,8-Cineole synthase	0.03	0.09	0	0.04	0.02
evm.model.LG06.2070	TPS-Cin	K07385	1,8-Cineole synthase	0.09333	0.63	0.01	0.00667	0.03667
evm.model.LG06.2071	TPS-Cin	K07385	1,8-Cineole synthase	0.05	1.78667	0.01	0.12	0.49667
**map00904 Diterpenoid biosynthesis**								
evm.model.LG03.1976	TPS04	K17982	Geranyllinalool synthase	16.7267	111.243	119.477	16.9167	3.21
evm.model.LG03.1977	TPS04	K17982	Geranyllinalool synthase	0.39667	0.03667	0.01333	0.00333	0
evm.model.LG03.1979	TPS04	K17982	Geranyllinalool synthase	0.21333	0.13	0.12	0.01	0.00333
evm.model.LG03.1983	TPS04	K17982	Geranyllinalool synthase	0.05667	0.03333	0.01333	0	0
evm.model.LG04.832	CYP82G1	K17961	Trimethyltridecatetraene/dimethylnonatriene synthase	248.93	212.477	115.817	25.2067	40.0133
evm.model.LG04.833	CYP82G1	K17961	Trimethyltridecatetraene/dimethylnonatriene synthase	55.6333	19.6767	3.82667	0.61333	1.1
evm.model.LG09.1491	CYP82G1	K17961	Trimethyltridecatetraene/dimethylnonatriene synthase	2.65667	0.89667	0.01667	0.02	0
**map00520 Amino sugar and nucleotide sugar metabolism**								
evm.model.LG07.1944	UGDH	K00012	UDP-glucose 6-dehydrogenase	156.793	348.167	614.247	376.23	251.697
evm.model.LG09.1402	UGDH	K00012	UDP-glucose 6-dehydrogenase	0.02667	0.08	0.00667	0	0
evm.model.LG10.1337	UGDH	K00012	UDP-glucose 6-dehydrogenase	1.12667	6.45333	0.52667	0.07667	0
evm.model.LG11.1458	UGDH	K00012	UDP-glucose 6-dehydrogenase	15.22	26.78	15.09	4.99667	6.13333
**map00500 Starch and sucrose metabolism**								
evm.model.Contig9.58	BGL	K01188	Beta-glucosidase	22.6267	57.1533	36.8933	26.1667	2.60333
evm.model.Contig9.61	BGL	K01188	Beta-glucosidase	59.76	21.3733	0.11667	0.15333	0
evm.model.Contig9.63	BGL	K01188	Beta-glucosidase	0	0	0.75333	0.21333	0.04
evm.model.Contig9.67	BGL	K01188	Beta-glucosidase	0.00667	0	0.03667	0	0
evm.model.Contig9.68	BGL	K01188	Beta-glucosidase	0	0	34.0667	13.4433	0.79667
evm.model.Contig9.69	BGL	K01188	Beta-glucosidase	0	0.01333	0	0	0
evm.model.Contig9.71	BGL	K01188	Beta-glucosidase	0.00667	3.87667	7.58333	5.32333	0.05333
evm.model.Contig9.72	BGL	K01188	Beta-glucosidase	0	0.06333	33.7133	61.4567	0.57333
evm.model.Contig9.73	BGL	K01188	Beta-glucosidase	0	0.02	0.62	19.13	3.03667
evm.model.Contig9.75	BGL	K01188	Beta-glucosidase	0.72	11.5667	0.26333	0.18667	0.62667
evm.model.Contig17.40	BGL	K01188	Beta-glucosidase	0.08333	0	0	0	0
evm.model.Contig17.41	BGL	K01188	Beta-glucosidase	151.553	818.143	349.103	68.2433	7.36
evm.model.LG01.1136	BGL	K01188	Beta-glucosidase	0	0	0	0	0
evm.model.LG01.1138	BGL	K01188	Beta-glucosidase	0.07333	8.79333	0.02333	0.16667	0.05
evm.model.LG01.1139	BGL	K01188	Beta-glucosidase	42.1533	54.42	0.08	0.00667	0.48
evm.model.LG01.1140	BGL	K01188	Beta-glucosidase	67.6	26.4133	0	0	0
evm.model.LG01.1544	BGL	K01188	Beta-glucosidase	187.957	98.37	18.32	8.98667	1.66667
evm.model.LG01.1546	BGL	K01188	Beta-glucosidase	69.1667	331.207	616.703	234.93	121.037
evm.model.LG01.1582	BGL	K01188	Beta-glucosidase	23.7533	119.967	179.72	168.71	139.02
evm.model.LG01.1585	BGL	K01188	Beta-glucosidase	248.147	91.5567	13.0033	3.62333	0.53667
evm.model.LG02.413	BGL	K01188	Beta-glucosidase	25.2	50.8533	26.1033	16.12	3.46333
evm.model.LG02.419	BGL	K01188	Beta-glucosidase	0	0	0.19667	0	0.07333
evm.model.LG02.423	BGL	K01188	Beta-glucosidase	0	0	0	0.01333	0
evm.model.LG02.424	BGL	K01188	Beta-glucosidase	0	0	35.5367	26.6967	1.50333
evm.model.LG02.425	BGL	K01188	Beta-glucosidase	0	0	0	0	0
evm.model.LG02.428	BGL	K01188	Beta-glucosidase	0.04	0.61	1.23	0.74333	0.06
evm.model.LG02.430	BGL	K01188	Beta-glucosidase	0.01	0.00667	30.8367	12.3067	9.07667
evm.model.LG02.432	BGL	K01188	Beta-glucosidase	0.01333	2.04667	202.103	118.817	5.81
evm.model.LG02.434	BGL	K01188	Beta-glucosidase	0.34	7.6	0.72	0.21667	0.61
evm.model.LG02.435	BGL	K01188	Beta-glucosidase	0	0.01667	0	0	0
evm.model.LG02.436	BGL	K01188	Beta-glucosidase	0	0.00667	0	0	0
evm.model.LG02.438	BGL	K01188	Beta-glucosidase	0	0.45667	0	0	0
evm.model.LG02.440	BGL	K01188	Beta-glucosidase	0	0	0	0	0
evm.model.LG02.441	BGL	K01188	Beta-glucosidase	13.2033	69.4567	1.65667	1.2	1.75333
evm.model.LG02.437	BGL	K01188	Beta-glucosidase	0	0.01	0	0	0
evm.model.LG03.1694	BGL	K01188	Beta-glucosidase	0.06667	0.45	3.50667	0.74667	0.21333
evm.model.LG04.261	BGL	K01188	Beta-glucosidase	0	0	0	0	0
evm.model.LG04.267	BGL	K01188	Beta-glucosidase	0	0.01333	0	0	0
evm.model.LG04.269	BGL	K01188	Beta-glucosidase	0	0.04667	8.26	50.92	0.79333
evm.model.LG04.270	BGL	K01188	Beta-glucosidase	0	0	0	0	0
evm.model.LG04.288	BGL	K01188	Beta-glucosidase	0	0	0	0	0
evm.model.LG04.282	BGL	K01188	Beta-glucosidase	0.83333	19.5467	30.5433	13.1633	1.83667
evm.model.LG04.284	BGL	K01188	Beta-glucosidase	1.51	24.0133	20.0967	0.32	0
evm.model.LG04.287	BGL	K01188	Beta-glucosidase	2.74	28.0533	4.49	58.27	7.89333
evm.model.LG09.375	BGL	K01188	Beta-glucosidase	5.27	8.46667	3.78667	2.01	4.29333
evm.model.LG10.1336	BGL	K01188	Beta-glucosidase	4.03	73.2367	87.4467	16.2667	3.22333
evm.model.LG03.1788	bglB	K05350	Beta-glucosidase	2.93667	7.08333	4.16333	5.34667	20.4833
evm.model.LG03.1789	bglB	K05350	Beta-glucosidase	0.22	2.81	2.54667	1.55667	0.66333
evm.model.LG09.434	bglB	K05350	Beta-glucosidase	95.9433	31.1733	16.6867	48.7467	43.5933
evm.model.LG09.435	bglB	K05350	Beta-glucosidase	2.18	37.3067	22.8067	52.3467	22.6467
evm.model.LG09.437	bglB	K05350	Beta-glucosidase	7.1	6.92667	3.42333	4.49667	2.14
evm.model.LG09.439	bglB	K05350	Beta-glucosidase	0.06	0.08	11.65	8.29333	2.06
evm.model.LG11.510	bglB	K05350	Beta-glucosidase	85.57	49.7933	3.48	0.54	0.16333
evm.model.LG11.511	bglB	K05350	Beta-glucosidase	0.41667	0.3	0.06667	0.01333	0.16333
evm.model.Contig39.4	TPS	K16055	Trehalose 6-phosphate synthase	15.4867	73.2933	43.9667	5	37.4367
evm.model.Contig39.5	TPS	K16055	Trehalose 6-phosphate synthase	3.91	21.4633	14.58	7.01333	15.7833
evm.model.LG01.1074	TPS	K16055	Trehalose 6-phosphate synthase	6.73667	34.4933	24.2433	18.17	28.1533
evm.model.LG03.2315	TPS	K16055	Trehalose 6-phosphate synthase	19.2967	101.067	92.2433	40.34	215.353
evm.model.LG08.1291	TPS	K16055	Trehalose 6-phosphate synthase	41.9	53.1867	42.44	52.96	67.69
evm.model.LG09.905	TPS	K16055	Trehalose 6-phosphate synthase	19.2167	26.2233	27.9833	11.9333	106.677
evm.model.LG10.643	TPS	K16055	Trehalose 6-phosphate synthase	31.3867	76.1733	40.0067	88.42	17.5333
evm.model.LG10.1589	TPS	K16055	Trehalose 6-phosphate synthase	5.46	33.8267	13.19	5.32333	1.20333
evm.model.LG01.1341	GN5/6	K19893	Glucan endo-1,3-beta-glucosidase 5/6	1.00333	8.35333	3.99333	6.90333	5.1
evm.model.LG08.125	GN5/6	K19893	Glucan endo-1,3-beta-glucosidase 5/6	3.35667	2.94	1.17333	5.68	9.58
evm.model.LG01.863	SPS	K00696	Sucrose-phosphate synthase	102.98	43.6567	33.88	52.98	63.7567
evm.model.LG05.1250	SPS	K00696	Sucrose-phosphate synthase	2.65333	0.06	0.02	0.00667	0.23667
evm.model.LG05.1262	SPS	K00696	Sucrose-phosphate synthase	1.14	0.00333	0.00667	0.00667	0.90667
evm.model.LG06.1362	SPS	K00696	Sucrose-phosphate synthase	14.24	11.4567	3.85	2.16	3.70333
evm.model.LG08.1766	SPS	K00696	Sucrose-phosphate synthase	0	0.09333	0.03333	0.11	0.14333
evm.model.LG06.913	AMY	K01176	Alpha-amylase	0.01333	0.16667	0.84333	1.1	0.39333
evm.model.LG06.914	AMY	K01176	Alpha-amylase	0.64667	1.53667	0.72333	1.19667	1.52333
evm.model.LG06.923	AMY	K01176	Alpha-amylase	3.34	5.70667	3.81333	1.76	3.73
evm.model.LG06.924	AMY	K01176	Alpha-amylase	1.73	1.76333	1.34	1.07	6.63
evm.model.LG03.433	sacA	K01193	Beta-fructofuranosidase	65.3167	361.78	322.96	407.55	372.833
evm.model.LG03.435	sacA	K01193	Beta-fructofuranosidase	5.29667	142.247	22.87	9.19	100.593
evm.model.LG08.847	sacA	K01193	Beta-fructofuranosidase	0.46	2.43	0.18667	0	0
evm.model.LG08.1872	TREH	K01194	Alpha, alpha-trehalase	16.1967	25.3167	16.1733	14.4	31.3667
evm.model.LG08.1875	TREH	K01194	Alpha, alpha-trehalase	17.9767	17.2033	11.24	15.68	9.10333

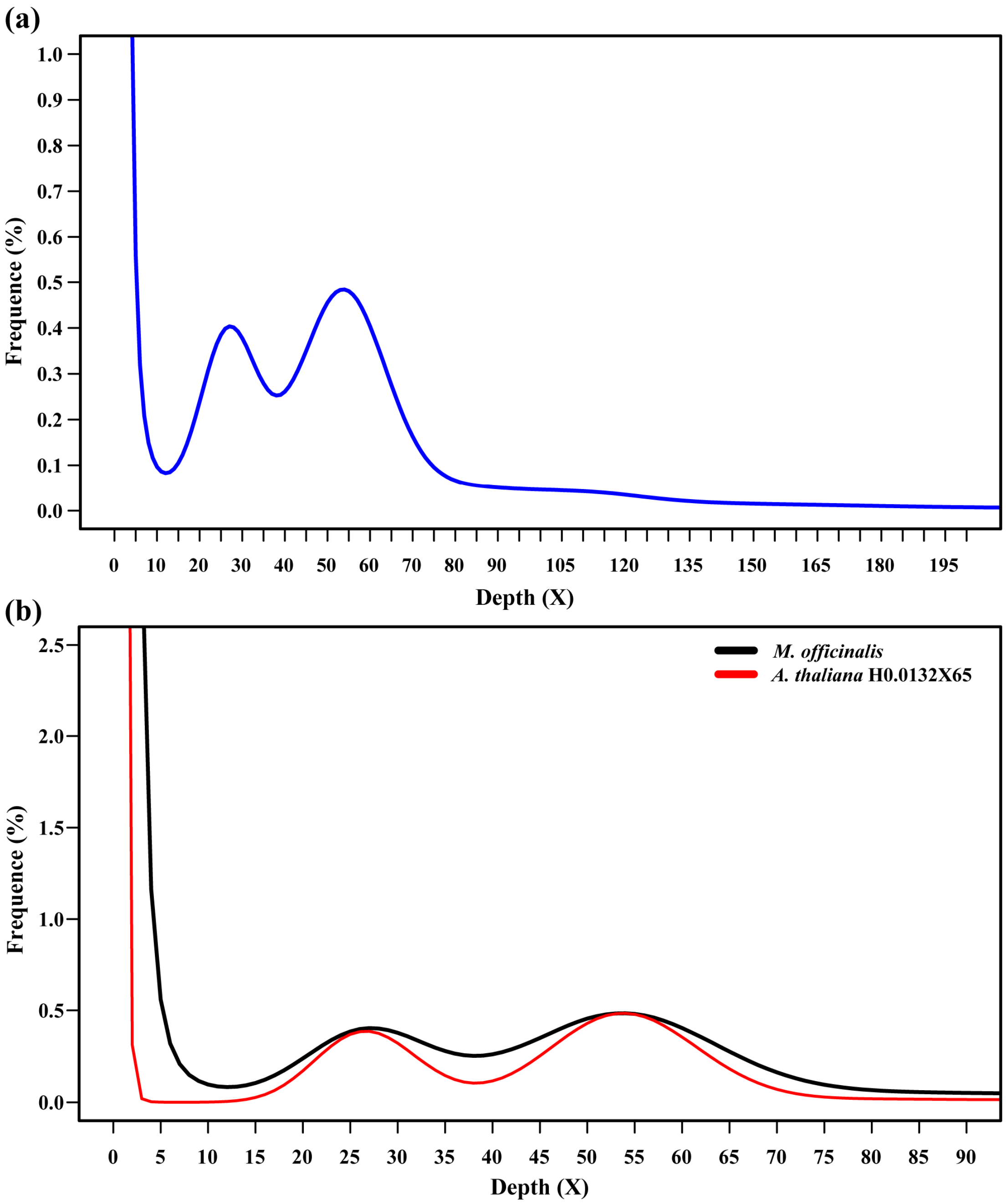

Supplementary Figure S1. Estimation of *M. officinalis* genome size by K-mer analysis. **(a)** The 17-mer frequency distribution of the genome survey. **(b)** Simulation curve of heterozygosity rate of *M. officinalis*.

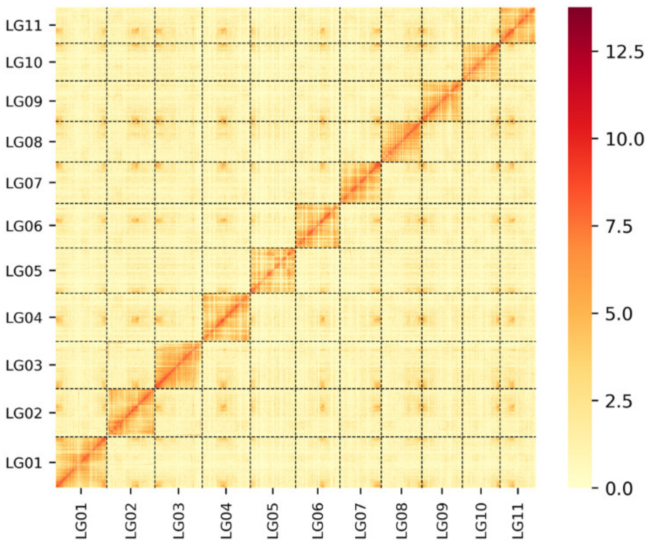

Supplementary Figure S2. The genome-wide all-by-all Hi-C interation heatmap of *M. officinalis*. LG01 ~ LG11 represent the 11 pseudochromosomes.

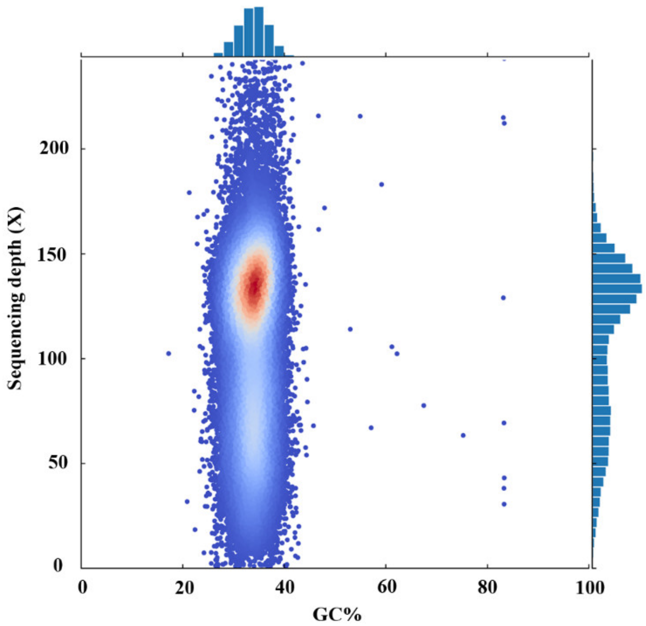

Supplementary Figure S3. The GC depth distribution of *M. officinalis* genome

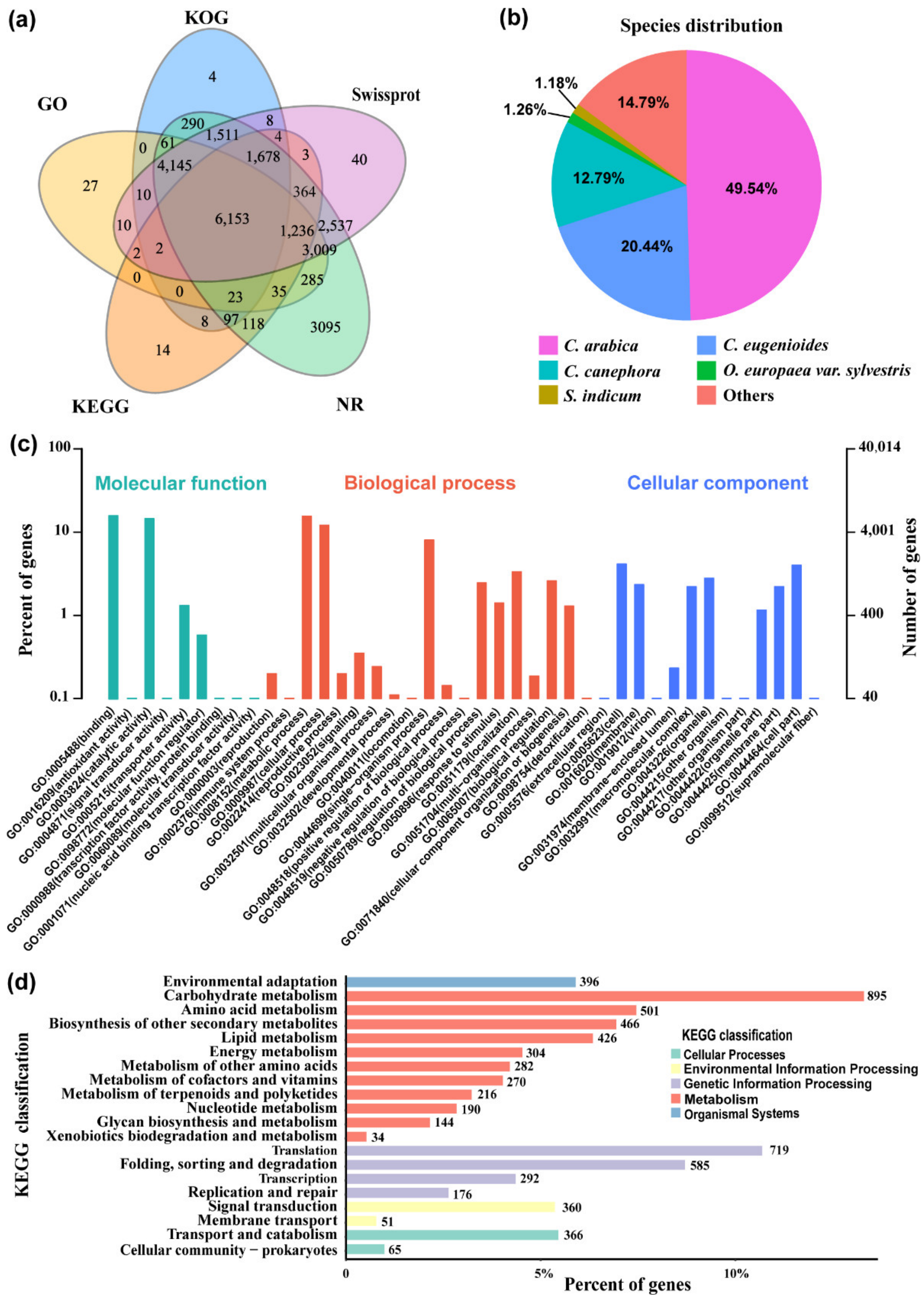

Supplementary Figure S4. Gene annotation in *M. officinalis*. (a) Summary of gene function annotation. Overall, 24,769 (91.39%) genes were functionally annotated at least one of the public databases. **(b)** Species distribution of the top BLAST hits. Species of the genus *Coffea* showed the highest proportion (82.77%) of homologous genes. **(c)** GO function classification. A total of 14,998 (55.34%) genes were categorized into three different groups. **(d)** KEGG function classification. 9,737 (35.93%) genes were annotated in the KEGG pathway database.

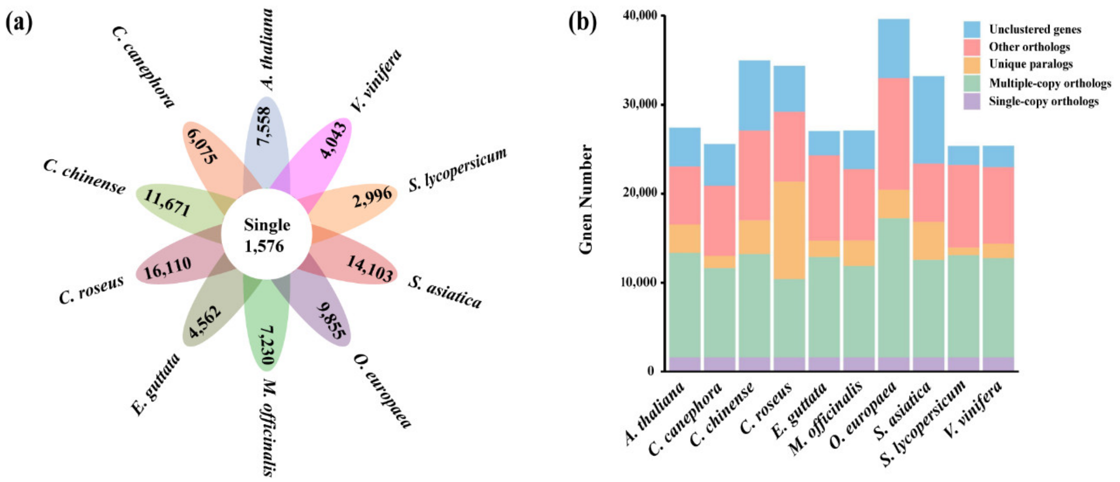

Supplementary Figure S5. The identified gene families of *M. officinalis*. **(a)** The shared and unique gene families of *M. officinalis* and nine other species. Single represents the single-copy genes and the numbers on the petals represent the number of species-specific genes. **(b)** Sumarry of the number of different gene family groups.

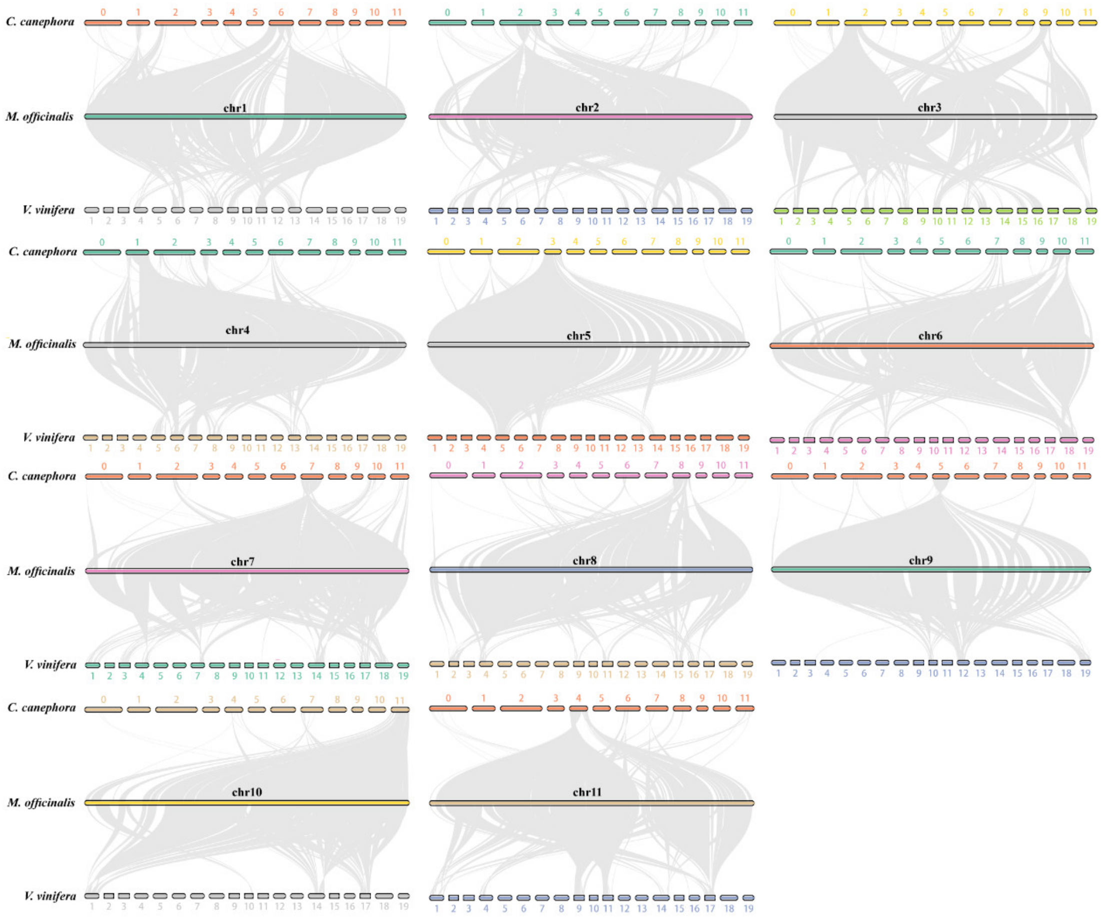

Supplementary Figure S6. Collinear relationship of *M. officinalis*, *C. canephora* and *V. vinifera*.

The gray line connects matched gene pairs.

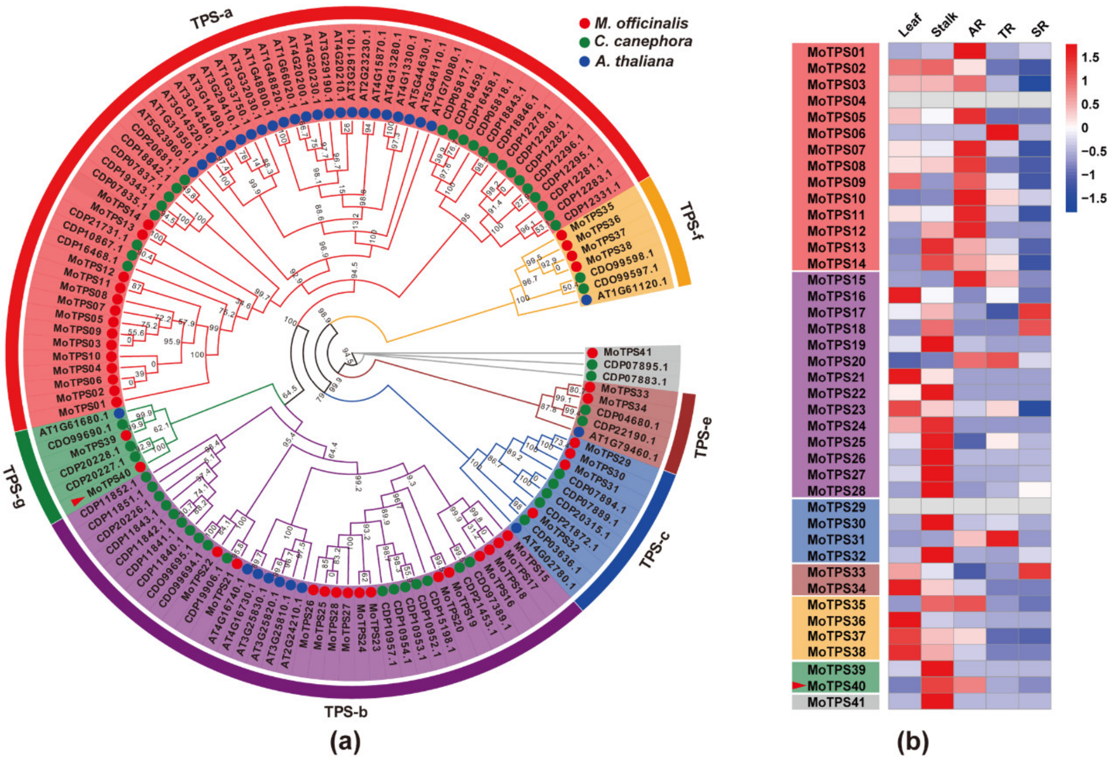

Supplementary Figure S7. Identification of TPS gene family in *M. officinalis*. **(a)** Evolutionary analysis of TPS gene family in *M. officinalis*. MAFFT software was used to perform multiple sequence alignment, conserved sequence was identified by Gblocks software and the maximum likelihood phylogenies were inferred using IQ-TREE under the model automatically selected for 5000 ultrafast bootstraps. **(b)** Expression patterns of TPS gene family in different tissues. The red triangle represents the GES gene.

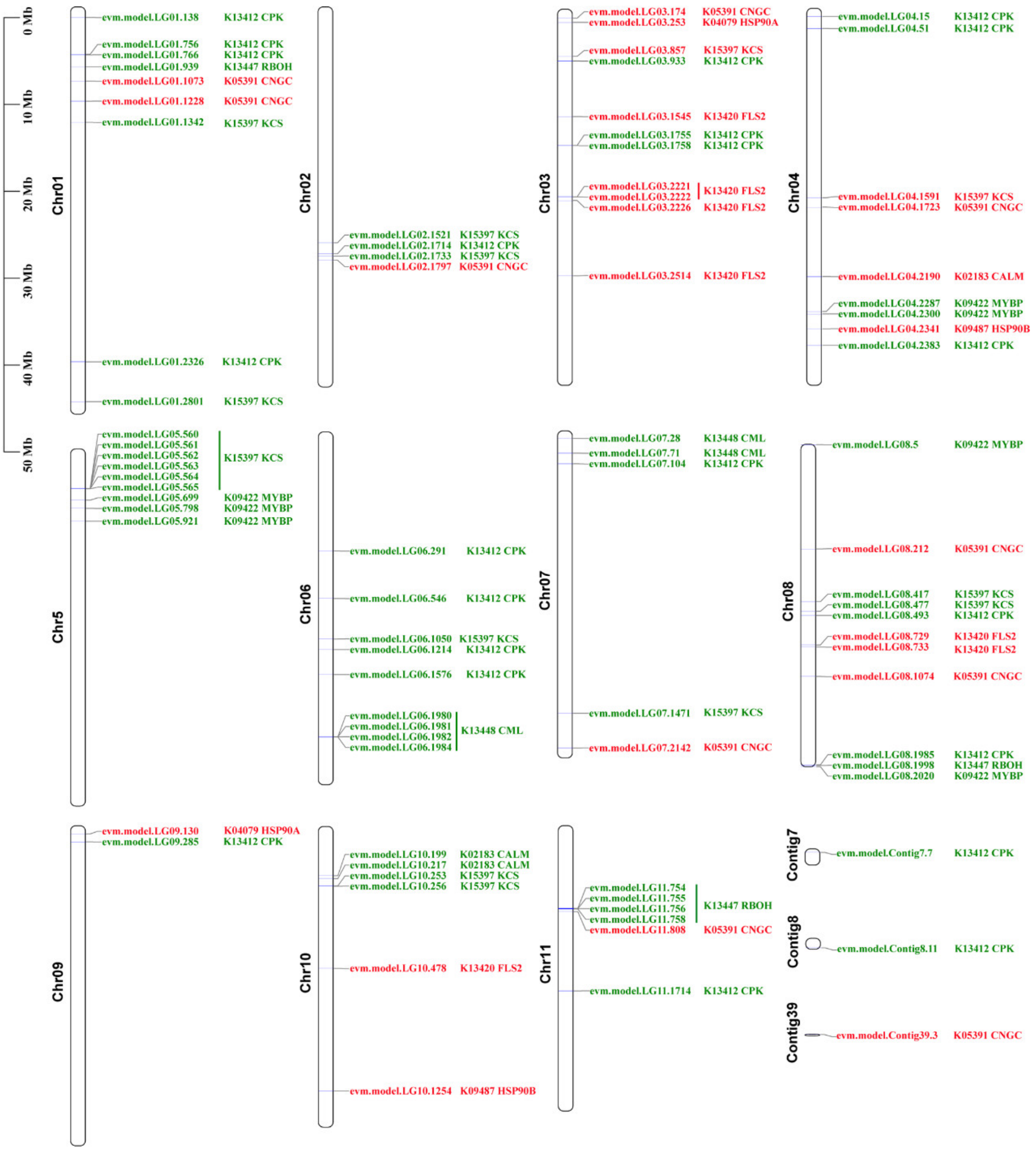

Supplementary Figure S8. Chromosomal locations of expanded gene families related to plant-pathogen interactions in the *M. officinalis* genome. Gene family expansion (green) and contraction (red).
